# Characterization of challenging forensic DNA traces using advanced molecular technologies

**DOI:** 10.1007/s00414-025-03448-8

**Published:** 2025-03-01

**Authors:** Amel Larnane, Caroline Lefèvre-Horgues, Corinne Cruaud, Cédric Fund, Edith Le Floch, Florian Sandron, Béatrice Segurens, Alexandre How-Kit, Jean-François Deleuze

**Affiliations:** 1 Institut de Recherche Criminelle de La Gendarmerie Nationale (IRCGN), 95000 Cergy-Pontoise, France; 2 Commissariat À L’énergie Atomique Et Aux Énergies Alternatives (CEA), Centre National de Recherche en Génomique Humaine (CNRGH), 91000 Evry-Courcouronnes, France; 3 Commissariat À L’énergie Atomique Et Aux Énergies Alternatives (CEA), Centre National de Séquençage (CNS), 91000 Evry-Courcouronnes, France; 4 Laboratory for Genomics, Foundation Jean Dausset – CEPH, Paris, France

**Keywords:** Forensic casework, DNA trace, Degraded DNA, Femto pulse system, *Alu* sequences, Microorganism analysis

## Abstract

The majority of crime scenes contain DNA that is either present in small amounts or degraded, making it difficult to obtain usable DNA profiles using conventional technologies. The current standard for analyzing casework samples is the specific amplification of short tandem repeats (STR), which is limited by DNA quality and quantity. Since the goal of forensic science is to identify a suspect or victim regardless of trace quality, we evaluated three technological approaches to better characterize and exploit these traces: (i) ultra-sensitive pulse-field electrophoresis on a Femto Pulse System (FPS) to visualize DNA content, (ii) real-time quantitative PCR based on *Alu* repeats to quantify human DNA and analyze its integrity, and (iii) 16S ribosomal RNA gene (16S rRNA) amplicon sequencing to identify microbiota. We optimized FPS analysis using DNA from model traces (blood, saliva, semen, touch DNA, and vaginal swabs) and applied the protocol to 100 casework samples. We found differences between the FPS profiles of model and casework samples, showing a variation in fragment size and distribution, suggesting the presence of non-human DNA. Using *Alu*-qPCR and 16S rRNA amplicon sequencing, we determined the amount and proportion of human and non-human DNA. Human DNA was detected in 84% of traces with an average of 70 pg per trace, while 16S rRNA revealed microbial DNA as the most abundant DNA in traces. These analyses provide new insights into forensic trace composition, allowing better sorting and profiling of traces.

**Supplementary Information:**

The online version contains supplementary material available at 10.1007/s00414-025-03448-8.

## Introduction

The smallest trace can become evidence at a crime scene, but some of these traces are not exploitable. In France, a DNA profile must contain at least six complete STR markers from the list defined in Article A38 of the Code of Criminal Procedure in order to be included in the national database. Therefore, it is essential to find a way to explore and understand their composition through optimized and dedicated analysis methods, especially to support DNA profiling [1, 2]. Since the majority of tissues found at crime scenes are either blood, saliva, touch DNA samples and semen [3], the field of forensic genetics has focused on studies and technologies that enable conclusive DNA profiling from these tissues [4]. However, a major limitation is that the environment (UV exposure, humidity, temperature, contamination by biological organisms, etc.) can alter these biological tissues and, in particular, the integrity of the DNA [5–8]. The main techniques used today are not optimized to exploit minute amount of DNA (of the order of a few picograms), which is a problem because the majority of traces found at crime scenes are touch DNA, which contains small amount of biological material. Consequently, almost two-thirds of all traces (according to IRCGN laboratory—the French Institut de Recherche Criminelle de la Gendarmerie Nationale—data) do not yield usable results (i.e. those suboptimal traces are either discarded directly after DNA quantification or because their DNA profile does not provide enough unambiguous genotypes to be included in the national database).

In forensic genetics, trace analysis is classically performed using a multiplex PCR that amplifies a set of highly polymorphic short tandem repeat (STR) markers. The applied protocol depends on the platform and the kit used, but generally allows for the analysis of fragments ranging from 100 to 400 bp. However, STR typing methods do not provide direct information about the fragmentation profile of the DNA, although the absence of the largest PCR fragments might indicate the presence of highly fragmented DNA in the sample [9, 10]. In recent years, real-time quantitative PCR (qPCR) techniques using a specific human-derived sequence allowed the quantification of human DNA fraction and also the determination of its fragmentation level [11]. However, these techniques have their own limitations, as they only assess a specific part of the genome and therefore cannot account for its overall fragmentation state. Thus, there is an urgent need for other technologies to assess global DNA integrity, a problem that has already been observed in other scientific fields where many developments are underway. In particular, developments in the analysis of ancient DNA [12], DNA from formalin-fixed, paraffin-embedded biopsies (FFPE) [13], or circulating cell-free DNA in biofluids [14], could provide methodological improvements and analytical information transferable to forensics to better estimate DNA fragmentation and quantity.

In forensic laboratories, the DNA content of traces is currently quantified but not visualized. One promising technology is the electrophoresis-based 2100 Bioanalyzer technology (Agilent), which provides visual and quantitative information on the amount and the size of the DNA present in a sample as well as its state of degradation [15]. However, this technology requires tens of picograms of DNA, which would consume most of the DNA found in suboptimal traces, restricting further downstream analyses. The latest evolution of this technology, the Femto Pulse System (FPS, Agilent), uses a pulsed field that provides a better detection limit for DNA and a better resolution of small and large fragments. By applying a pulsed field instead of a continuous field, this new instrument gains a factor of 15 to 20 in separation speed, easily achieving 100 times higher sensitivity and allowing the analysis of samples down to the femtogram level [16]. FPS is used to analyze plasma cell-free DNA which is similar to DNA found in suboptimal traces due to its high fragmention level and very low concentrations [17]. To our knowledge, femtosecond pulse analysis has never been used for forensic samples. Thanks to its increased analytical sensitivity, FPS allows the assessment of the quantity and integrity of DNA from a suboptimal trace.

Another issue encountered when working with suboptimal traces found at crime scenes is the state of contamination by DNA from sources other than the person involved in the trace, including DNA of non-human origin such as animals, plants, bacteria etc. Since *Alu* sequences are the most abundant repeated sequences and are distributed throughout the human genome, their use should allow the estimation of the fraction of human DNA, as well as the determination of DNA integrity by amplifying fragments containing *Alu* sequences of different sizes [18]. The only limitation of this approach is that *Alu* repeats are found in other primate genomes [19, 20]. However, in the unlikely event that a trace could be contaminated with non-human primate DNA (crime scene in a circus, a zoo or in a laboratory, etc.), the result will be an overestimation of the proportion of human DNA, but might not affect the STR-PCR DNA profile results, as the sequences used for the analysis are human-specific. In most cases, the forensic scientists do not face with this type of problem, and therefore *Alu* sequences are suitable for studying the quantification and analysis of human DNA degradation from forensic traces [21].

On the other hand, analysis of the contaminants that can interfere with the analysis of the traces found at crime scenes can be useful in forensic. In fact, microorganisms are present in all types of environments, whether domestic, public or natural and, depending on the origin, in the trace itself. Again, forensic science can benefit from all the technologies recently developed to analyze the micro-organisms present in different human microbiota, whose role in the development of human diseases is now regularly studied. Their study is also growing in forensic science for several years [22, 23]. More specifically, the use of NGS technologies is a solution to analyze the microorganisms present in a casework sample, where the MiSeq sequencer (Illumina) is widely used [24]. The analysis of these organisms can therefore be an additional source of information to understand the composition of a trace found at a crime scene.

Therefore, understanding the main characteristics of a forensic DNA trace, is a complex subject, but necessary for the development of future technologies and their impact on DNA profiling, if we are to increase the number of traces usable in a national database. This article describes the use of different technologies to characterize forensic traces regardless of their composition, contamination and state of degradation using ultra-sensitive molecular electrophoretic technologies, real-time qPCR and NGS.

In summary, the aims of our study was to finely characterize the composition of suboptimal traces in order to 1) enable traces to be sorted according to their composition, and 2) use this knowledge in the future to adapt existing technologies or incorporate new ones (such as miniaturization and next-generation sequencing—NGS) to facilitate their genetic profiling. In this context, we will:

- Evaluate whether a high-resolution technology such as Femto Pulse could be used to obtain electrophoresis profiles for sorting traces according to their quantitative and qualitative composition in DNA.
- Evaluate whether *Alu*-qPCR could improve the prediction of traces that do not produce DNA profiles, by determining if they contain no human DNA or if they contain insufficient DNA or DNA degraded at a level not compatible with classical technologies.
- Analyze microbial contamination of traces, which has often been mentioned but never thoroughly characterized, and determine whether this contamination could serve as an additional signature in forensic analysis.

## Materials and methods

### Samples

Two sets of samples were used in our study: i) model samples, and ii) suboptimal casework samples.

#### Model samples

The model samples consist of a prospective collection designed to reproduce the majority of tissue types classically processed in a forensic laboratory, i.e., blood, saliva, touch DNA, semen, and vaginal swabs. Samples were collected from six donors—three males and three females—who signed an informed consent document established and validated by the IRCGN. Specific protocols were developed to increase the diversity of the model samples and to ensure that the tissues were collected under conditions similar to those encountered at crime scenes. For blood, three protocols were established. First, the blood was collected directly from a needle onto a swab. Second, donors placed a drop of blood on a cloth representing a bloodstain found on a piece of clothing belonging to another donor, in order to mimic the background noise of the traces coming from the clothing. Third a drop of blood was placed on a knife blade that had been cleaned to avoid external contamination. For saliva, samples were collected from the spit of two donors, the cutting of the ends of two cigarette butts (per donor) after the donors had smoked according to their habits, as well as the wiping of bottle necks after the donors had drunk 5-s sips at 1-min intervals (2 bottles per donor). Semen samples collected from both donors was diluted 1:5 before performing a 50 µl extraction. Vaginal swabs were collected upon the donors’ awakening by rotating the swabs for 10 s. Each swab was collected independently by the donors in the same manner. For touch DNA samples, four household knives were used and decontaminated (blade and handle). The donors then held one knife handle in each hand for 1 min. All deposits were air-dried for 4 h and then swabbed. All samples were then processed using exactly the same protocols as those applied to the casework samples before being analyzed in the forensic laboratory.

#### Suboptimal casework samples

The second type of samples corresponded to 100 touch DNA traces from real forensic cases (e.g., burglary, assault etc.) in which the DNA was present in very small quantities (< 100 pg) and did not yield usable DNA profiles using current laboratory methods (see Materials and methods, section 2.2). Various approvals were obtained to use these traces in this study, including approval from an ethics committee (Judicial Center of the National Gendarmerie), the signing of a confidentiality agreement (between the French National Gendarmerie and the National Center for Research in Human Genomics (CNRGH)) and finally, the approval of the data manager of the French National Gendarmerie.

### DNA extraction and STR typing by capillary electrophoresis fragment analysis

DNA from all samples was extracted using Crime Prep Adem-Kit (Ademtech) for casework with an extraction volume of 50 µl and assessed using the technologies described below. DNA samples were quantified using the Qubit™ dsDNA HS assay Kit on a Qubit 3.0 fluorometer (Thermo Fisher Scientific) according to the manufacturer’s instructions.

For STR typing, both sets of DNA samples were amplified using either the GlobalFiler™ Kit (Thermo Fisher Scientific) or the Investigator® 24plex QS Kit (Qiagen) using the standard protocols provided by the manufacturers, including 30 PCR cycles. The amplicons were prepared for fragment analysis by capillary electrophoresis (CE) using an ABI 3500xl Genetic Analyzer (Thermo Fisher Scientific). 1 μl of the PCR product (or GlobalFiler™/ Investigator® 24plex allelic ladder) was mixed with 10 μl of formamide / size standard solution (Thermo Fisher Scientific). Prior to electrophoresis, the samples were denatured at 95 °C for 3 min then chilled on a cold block for 3 min. Electrophoresis conditions were as follows: 24 s of injection at 1.2 kV and migration at 13 kV for 1550 s. CE results were analyzed using GeneMapper™ ID-X Software v1.6 (Thermo Fisher Scientific), with a threshold for allele peak calls set at 50 relative fluorescence units (RFU). This threshold is commonly used at IRCGN and is widely accepted in the forensic scientific community. It has been chosen to balance sensitivity and specificity in allele detection, ensuring that background noise or low-level artifacts do not compromise the reliability of the genetic profile, while maintaining the ability to detect true alleles.

### DNA integrity analysis using high sensitivity pulsed-field electrophoresis (FPS)

The quality of DNA samples, model and suboptimal casework samples, was assessed using the FPS (Agilent) to visualize their degradation state (amount and fragmentation). The FPS is based on a pulsed field, which allows to increase the separation speed and to reach a limit of detection in the low femtogram range.

#### Analysis kits

Two different kits were used for the analysis of the five different types of model samples: 1) the Ultra Sensitivity NGS Kit (Agilent) and 2) the Genomic DNA 165 kb Kit (Agilent), according to the conditions defined by the supplier. The two kits enable the analysis of DNA from different size ranges. The first kit has a reliable size range from 100 to 6 000 bp and the second from 1.3 to 165 kbp. The Ultra Sensitivity NGS Kit presents an error rate of about 5%, whereas the Genomic DNA 165 kb has an error rate of about 15% for DNA sizing, according to the manufacturer.

#### Optimization of the experimental parameters

In order to obtain the signal from the smallest amounts of DNA, different parameters of the standard FPS protocol were modified and optimized using the Ultra Sensitivity NGS Kit: (i) the separation parameters using three different time points (50 min, 70 min, and 100 min); (ii) the ladder and marker diluent reagent concentrations (1:5 and 1:20 dilutions) to improve the signal image over a range of DNA concentrations; (iii) the time and voltage injection parameter, where the applied injection time was tested between 30–60 s at 3, 5, or 10 kV; and (iv) the final reaction volume (from the standard condition of 20 µl, down to 12 µl, 10 µl, 8 µl, and 6 µl) to concentrate the sample.

The data obtained from the FPS were analyzed using the Prosize software provided by the supplier (Agilent). The software provides digital data for visual analysis of DNA samples and reports on different metrics such as size and concentration.

### Quantification and analysis of fragmentation rate of human DNA by *Alu*-qPCR

Real-time qPCR of *Alu* sequences has been used to quantify human genomic DNA. We used the primers [25] from the Swift 2S™ Turbo v2 DNA Library Kits: Input DNA Protocol (Integrated DNA Technologies—Swift Biosciences™), which include primers that amplify two different amplicons: one short (115 bp; *Alu115*) and one long (247 bp; *Alu247*) with the iTaq enzyme in the Universal SYBR Green Supermix (Bio-Rad). All reactions were performed on a Light Cycler 480 (Roche) according to the cycling program recommended by Swift Biosciences™.

The Input DNA Quantification Assay protocol (Swift Biosciences™) [25] was also adapted for samples containing very low amounts of DNA, using a serial dilution of human CEPH control DNA (NA12878) ranging from 1 ng to 0.002 pg (1 ng, 100 pg, 10 pg, 1 pg, 0.1 pg, 0.01 pg, 0.002 pg). Absolute DNA quantification from *Alu115* and *Alu247* PCR assays was performed using a standard curve based on these serial dilutions. Each point of the serial dilution was tested in duplicate, and the samples were run in triplicate.

The primate specificity of the method used was assessed by testing several DNA samples from other species, including three bacterial strains (at 1 ng/µl) and one macaque DNA (at 100 pg/µl) provided by the french National Sequencing Center (CNS) and the CNRGH. H_2_O and low Tris–EDTA were used as negative controls.

qPCR data were analyzed using the LightCycler 480 software 1.5.1 (Roche) which provides an estimate of the DNA concentration of the samples for each amplification separately. The *Alu115* and *Alu247* PCR results allow the calculation of a DNA fragmentation rate defined as *Alu115*/*Alu247* ratio. An integral DNA presents a score of 1, while low-quality DNA has a score greater than 1. The more degraded the DNA, the higher the score.

### Analysis of the presence of microorganisms in the casework samples

Amplicon sequencing of bacterial 16S ribosomal RNA (rRNA) was performed on six selected casework samples to identify the presence of microorganisms in the DNA traces. Two methods were employed [26]. The first method (Fuhrman method) targeted the V4 and V5 regions using 15F (5′- GTGYCAGCMGCCGCGGTAA-3′) and 926R (5′- CCGYCAATTYMTTTRAGTTT-3′) 16S primers. The second method (Nested method) involved an initial amplification of the entire 16S rRNA using 27F (5′- AGAGTTTGATCMTGGCTCAG −3′) and 1492R (5′- TACGGYTACCTTGTTACGACTT −3′) 16S primers, followed by a secondary amplification of the V4 and V5 regions with 15F and 926R 16S primers. A positive control (*Acinetobacter* bacteria) and two negative controls (H_2_O) were included.

The PCR products were end-repaired, A-tailed at the 3’end, and ligated to Illumina-compatible adaptors using the NEBNext DNA Modules Products (New England Biolabs) and NEXTFLEX Unique Dual Index Barcodes (Perkin Elmer). After two consecutive 1 × AMPure XP cleanups, the ligated products were amplified using the Kapa Hifi HotStart NGS Library Amplification Kit (Roche), followed by 1X AMPure XP purification. All libraries were quantified by Qubit dsDNA HS Assay measurement (Thermo Fisher Scientific). A size profile analysis was performed using a 2100 Bioanalyzer (Agilent). Libraries were sequenced using 2 × 250 bp paired-end sequencing on the Miseq System (Illumina).

Demultiplexing and FastQ generation were performed using the Illumina Local Run Manager, and further analysis of sequencing data was performed with R using an in-house developed script. The sequences were first grouped into clusters with the corresponding coverage of the identified bacterial species/eukaryotes. The proportion of bacteria and eukaryotes was then calculated for each sample. Then, two rankings were created in descending order according to the proportion of bacterial species and bacterial genus. Only the results for the bacterial genus were presented. These rankings were generated for all the samples and for the two methods: Nested and Fuhrman.

## Results

**Fig. 1:**
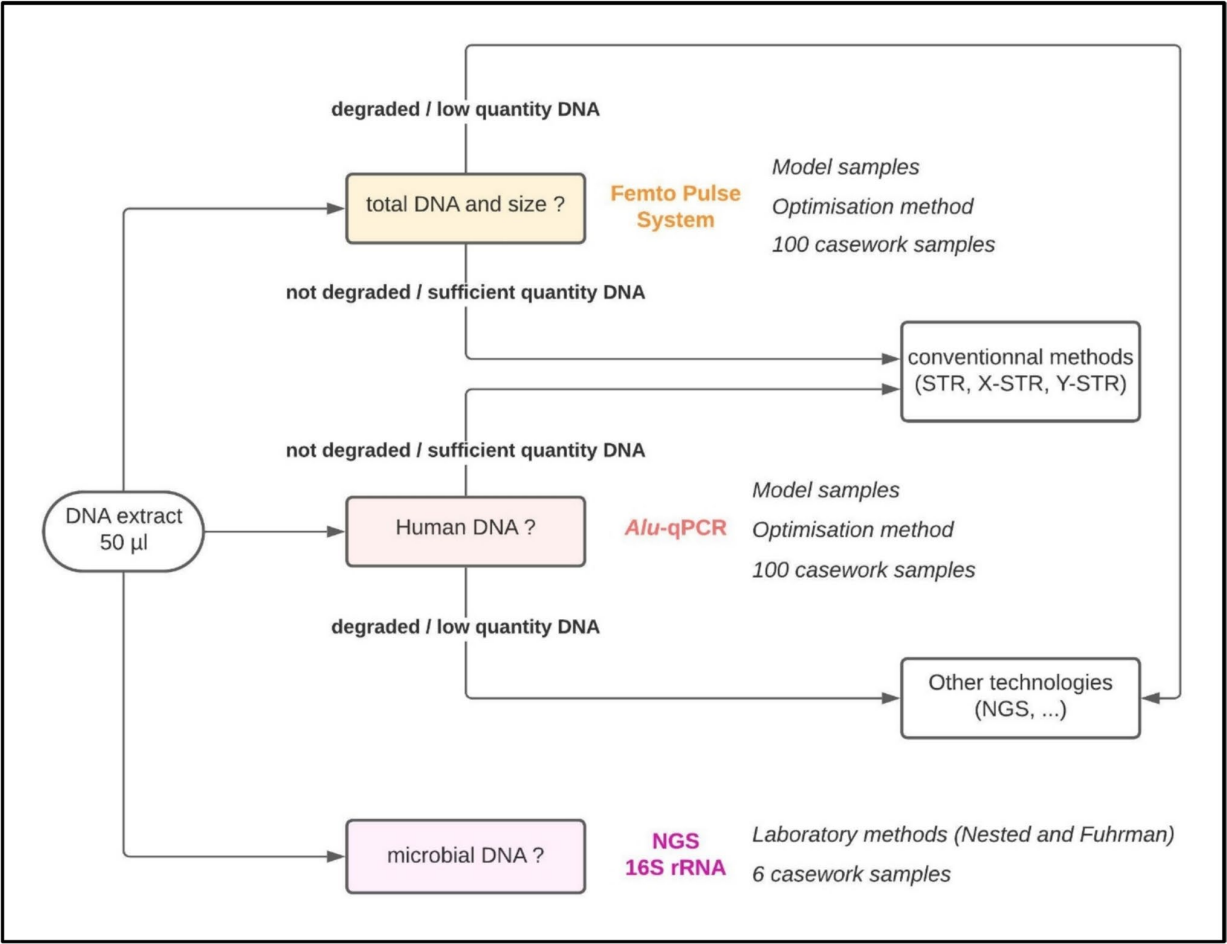
Description of the experimental workflow and methodological approaches used in our study

Our study aimed to characterize thoroughly forensic DNA samples that could be found at crime scenes using a panel of different molecular methods that are not typically used in routine forensic identification of individuals (Fig. 1). First, we sought to quantify and characterize the fragmentation profile of model DNA samples as well as 100 suboptimal casework samples using highly sensitive pulse-field electrophoresis, which enables the sensitive detection of DNA at high resolution. This required several optimization steps to make this approach fully compatible with trace samples. Next, *Alu*-qPCR was quantified the amount of amplifiable human DNA and evaluated its integrity on 100 casework samples. Finally, a metagenomic analysis of the different species present in the DNA samples was performed by amplicon sequencing of 16S rRNA using two methods known as the Nested and Fuhrman methods. These complementary analyses help in the selection of conventional methods (e.g., STR, X-STR, Y-STR genotyping) when the DNA is not too degraded and there is sufficient human DNA, or alternative technologies, such as NGS for the analysis of other genetic markers, for highly degraded samples or those with limited human DNA.

### DNA trace degradation analysis using the FPS

#### FPS profiles of the model samples using two different kits

We first analyzed the FPS protocol using model samples representative of those found at a crime scene. DNA was extracted from the 28 model samples from 6 donors and we obtained DNA concentrations that varied according to tissue type and donor, as represented in the dotplot in Supplementary material S1: between 0.02 ng/µl and 1.4 ng/µl for blood depending on the deposit support (swab, knife or clothing), between 0.02 ng/µl and 1.3 ng/µl for saliva (spit, bottle neck, cigarette butt); about 0.05 ng/µl for touch DNA samples; between 3 ng/µl and 9 ng/µl for semen; between 11 ng/µl and 14 ng/µl for vaginal swabs. All the samples collected were used for STR typing, and we obtained complete STR DNA profiles for almost all samples, except for one touch DNA from a donor, which had a partial DNA profile. The STR profiles obtained from a same donor were identical regardless of the tissue of origin and the support of the traces collected.

**Fig. 2:**
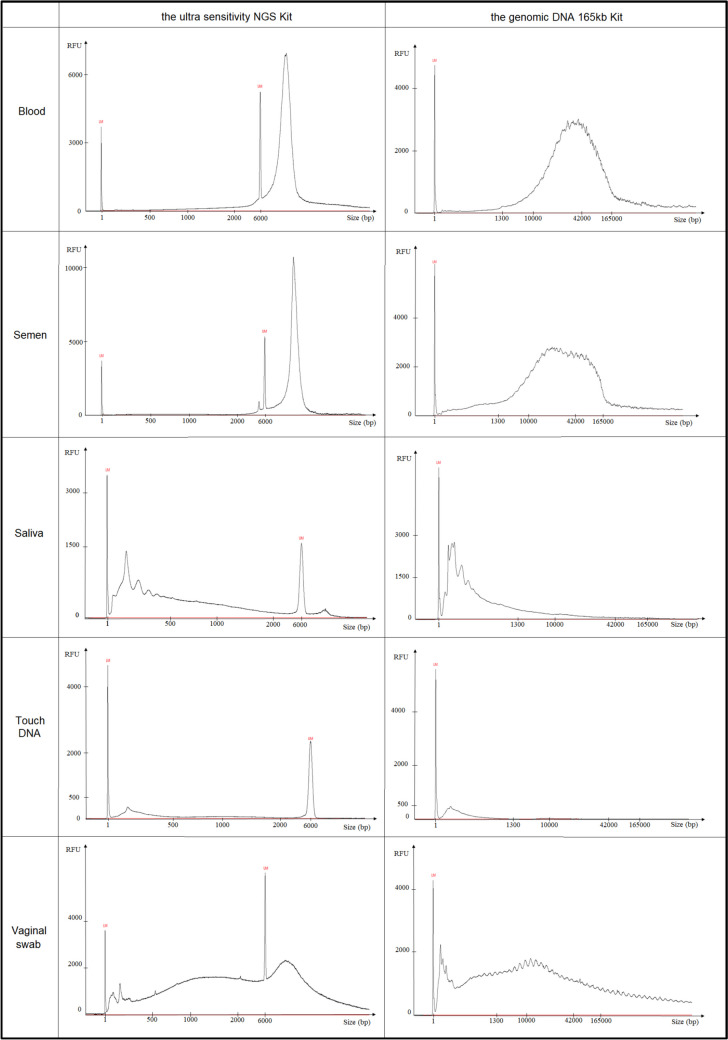
Electrophoregrams of DNA from various tissues (blood, semen, saliva, touch DNA, and vaginal swab) using the FPS reveal tissue-specific profiles. The Ultra Sensitivity NGS Kit uses two size markers (1 bp, lower marker (LM) and 6 000 bp, upper marker (UM)), while the Genomic DNA 165 kb Kit uses only one size marker (1 bp, lower marker (LM))

These samples were further used to evaluate the quantity and quality of DNA recovered using the FPS. The FPS profiles obtained are shown in Fig. 2, which presents a single support by tissue for the model samples (the knife for blood and the bottle neck for saliva). Results from other supports for blood and saliva for each donor are presented in Supplementary Material S2. Evaluation of both the Ultra Sensitivity NGS and the Genomic DNA 165 kb kits gave similar results, with the Ultra Sensitivity NGS Kit being more accurate and sensitive for small molecular weigtht DNA.

**Fig. 3:**
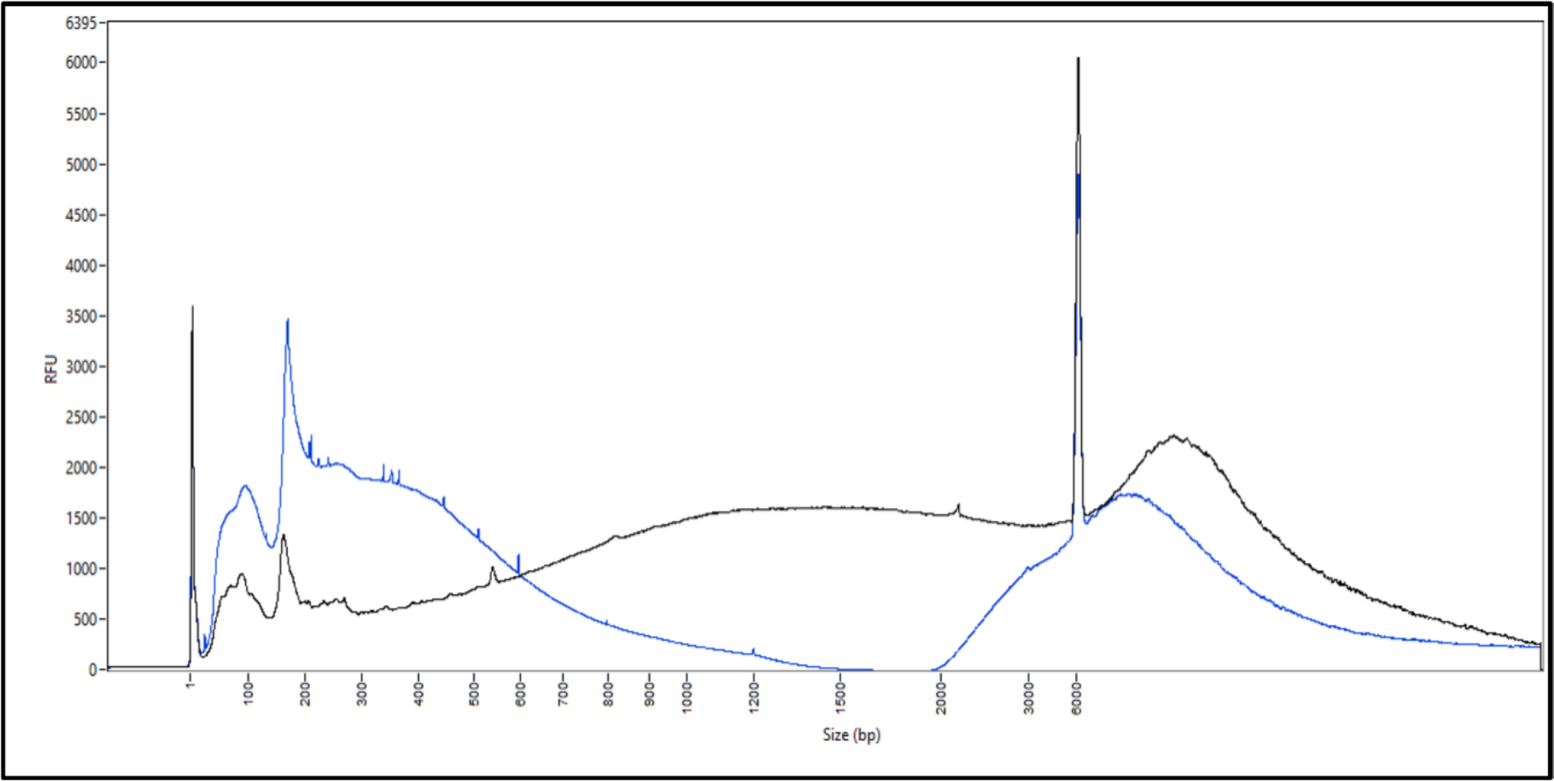
Femto pulse electrophoresis profiles of DNA from vaginal swabs from two donors (donor 1 in black and donor 2 in blue) revealing donor-specific profiles. The amount of DNA relative to size differs between the two donors

FPS analysis distinguishes three categories of profiles that may result from the tissue’s exposure to the external environment, but also from the specific individual metagenome associated with it in the human body:

- Category 1 (blood and semen): large DNA (> 6 000 bp) naturally isolated from the external environment, stable profile according to donor and tissue, rich in DNA;
- Category 2 (saliva and touch DNA): mainly small size DNA (< 500 bp), naturally exposed to the external environment, stable profile according to donor and tissue, poor in DNA;
- Category 3 (vaginal cells): exposed to the external environment, the FPS profile obtained is a mixture of the categories 1 and 2, and varies greatly depending on the donor, rich in DNA. An overlay of the two donors’ results for this tissue is shown in Fig. 3.

#### Optimization of the experimental parameters

**Fig. 4:**
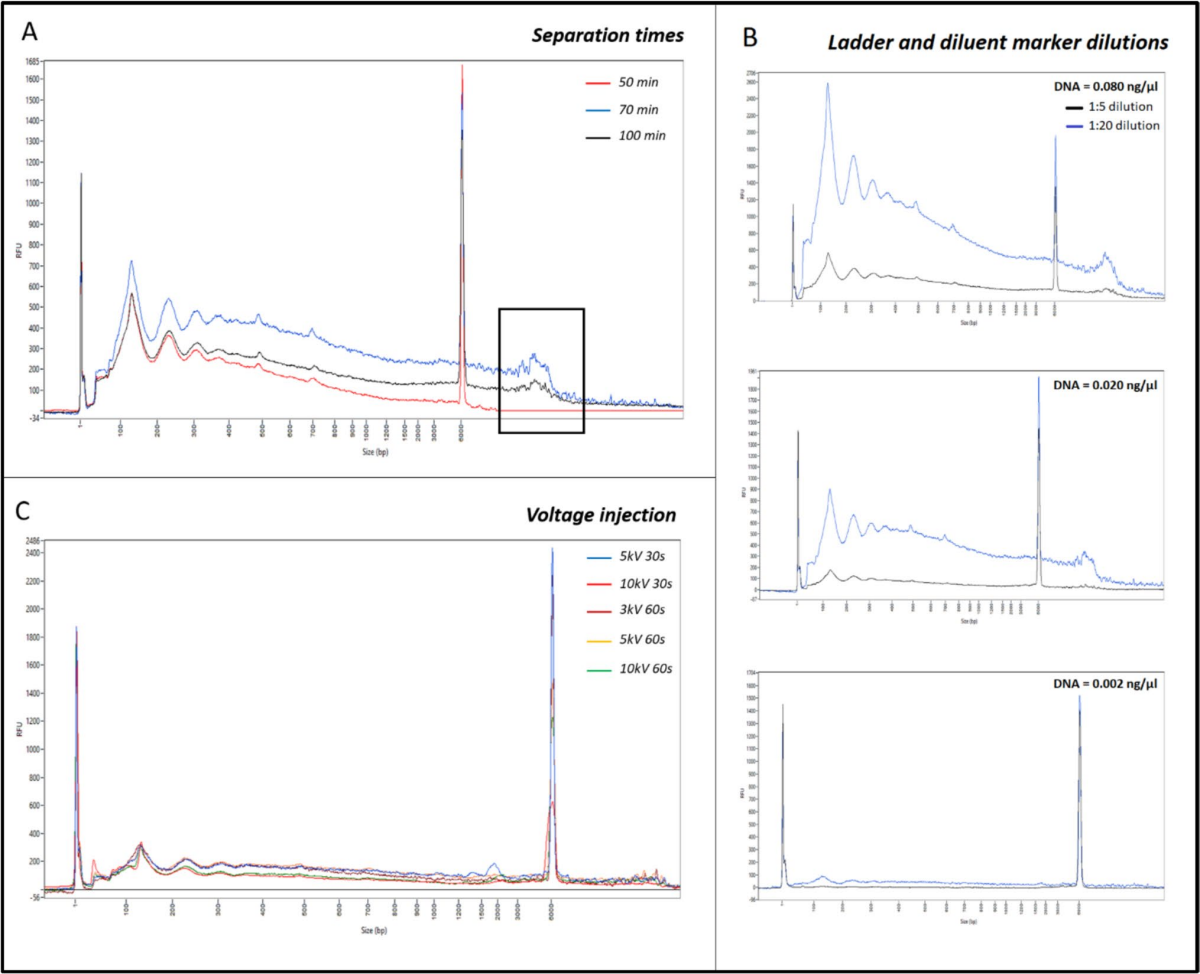
Femto pulse electrophoretic profiles of saliva DNA under different tested parameters.  Results obtained with (**a**) different separation times. The black square highlights DNA fragments above 6,000 bp, which were not detected at 50 min but were fully visualized at 70 and 100 min, **b** different ladder and diluent marker reagent dilutions, and (**c**) different voltage injections

We optimized several parameters on the FPS using DNA isolated from one saliva sample using decreasing concentrations ranging from 0.080 ng/µl to 0.002 ng/µl. The results are shown in Fig. 4. The saliva sample had a FPS profile similar to the touch DNA sample and contained more DNA, allowing for multiple optimization experiments.

First, we wanted to optimize the ‘separation’ time (Fig. 4a). We set the ‘Perform Prerun’ parameter at −3 kV for 2700 s [16] and tested different ‘separation’ times: 50 min, 70 min, and 100 min at −3 kV (voltage recommended by the supplier). The 50 min separation time did not allow the analysis of the whole sample, especially fragments larger than 6 000 bp. In contrast, there was no significant difference in resolution between 70 and 100 min, which allowed whole sample analysis. Therefore, we selected a time of 70 min for all subsequent analyses, which allowed the analysis of the whole DNA sample while reducing the duration of experiments by 30 min and maintaining the best resolution.

We then tested different concentrations of the ladder and diluent marker reagents to improve the visibility of the signal (Fig. 4b). We observed better results when using a 1:20 dilution of the two reagents compared to the 1:5 dilution. Certain characteristics of the sample were no longer visible at a concentration of 0.020 ng/µl with a 1:5 dilution. However, even at a DNA concentration of 0.002 ng/µl, the short fragments were still visible at a 1:20 dilution.

We further varied the strength and the time of the ‘sample voltage injection’ (Fig. 4c): 3, 5, and 10 kV for 30 s and 60 s (at a concentration of 0.006 ng/µl). The results were not different for our application, so we set the parameters to 5 kV for 30 s as recommended by the supplier.

Finally, the total volume per well was tested (12 µl, 10 µl, 8 µl, and 6 µl) while keeping the same amount of reagent and DNA. Results are presented in Supplementary material S3. The best results were obtained with a total volume per well of 10 µl; below this volume, the system cannot accurately pipette the sample. In addition, 10 µl allowed the sample concentration to be increased, which is an important consideration when analyzing suboptimal casework samples.

#### Analysis of suboptimal casework samples

We analyzed 100 suboptimal casework samples under optimal FPS conditions: 5 kV 30 s, 70 min, 1:20 ladder and diluent maker dilutions, and 10 µl final volume. These samples corresponded to touch DNA that yielded very low amounts of DNA (< 100 pg) and did not result in a usable DNA profile when applying conventional methods.

**Fig. 5:**
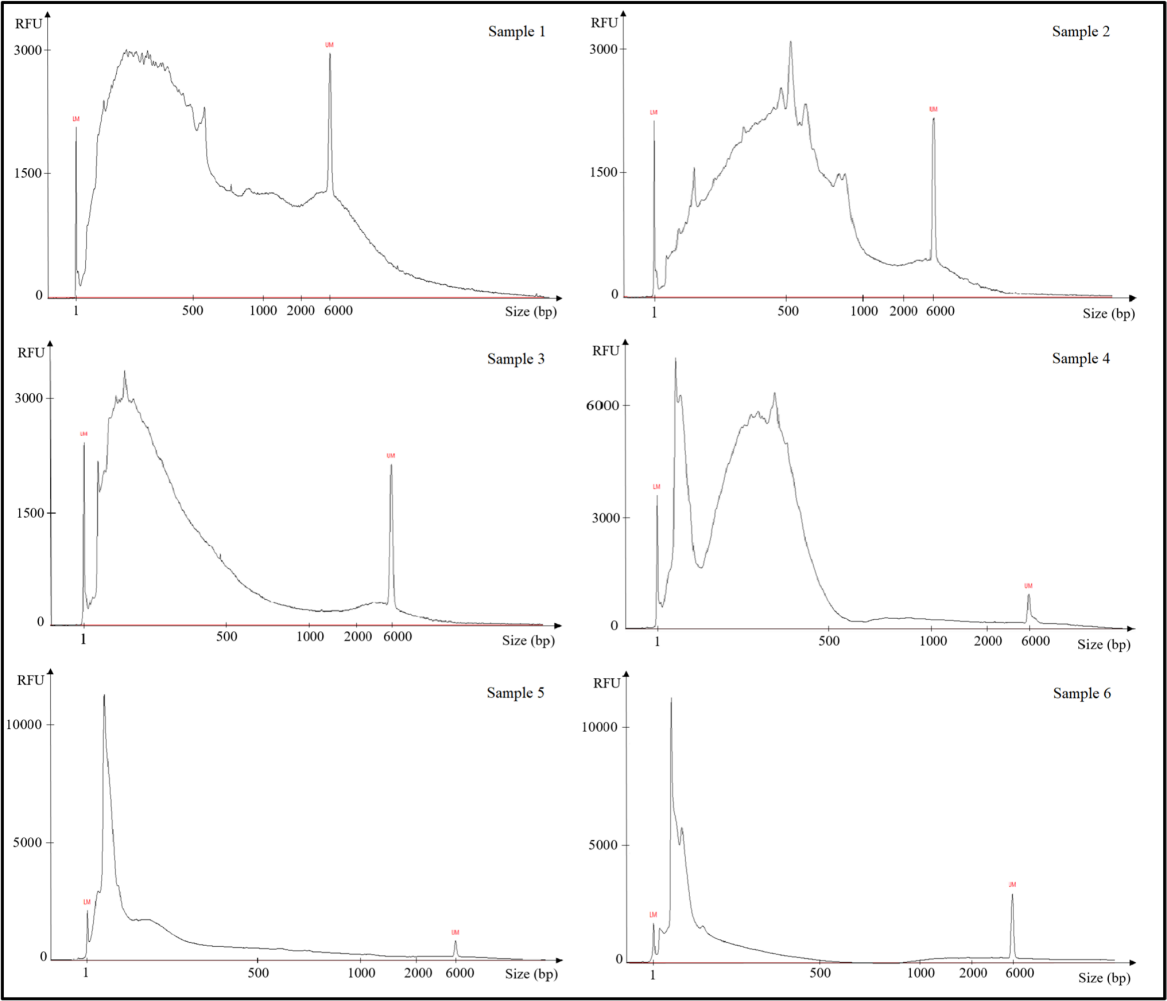
Femto pulse electrophoretic profiles of six casework samples representing ‘least degraded’ DNA profiles (samples 1 and 2), ‘moderately degraded’ profiles (samples 3 and 4), and profiles with degraded DNA (samples 5 and 6). The results show the diversity of FPS profiles obtained from the 100 touch DNA casework samples

The results obtained were very heterogeneous and in the majority of cases the profiles did not correspond to the Category 2 profile corresponding to a “fresh” touch DNA collected under controlled conditions (see Fig. 2). Representative examples of the FPS profiles obtained with touch DNA casework samples are shown in Fig. 5, illustrating varying levels of degradation, from ‘least-degraded’ to ‘highly degraded’ DNA.

**Fig. 6:**
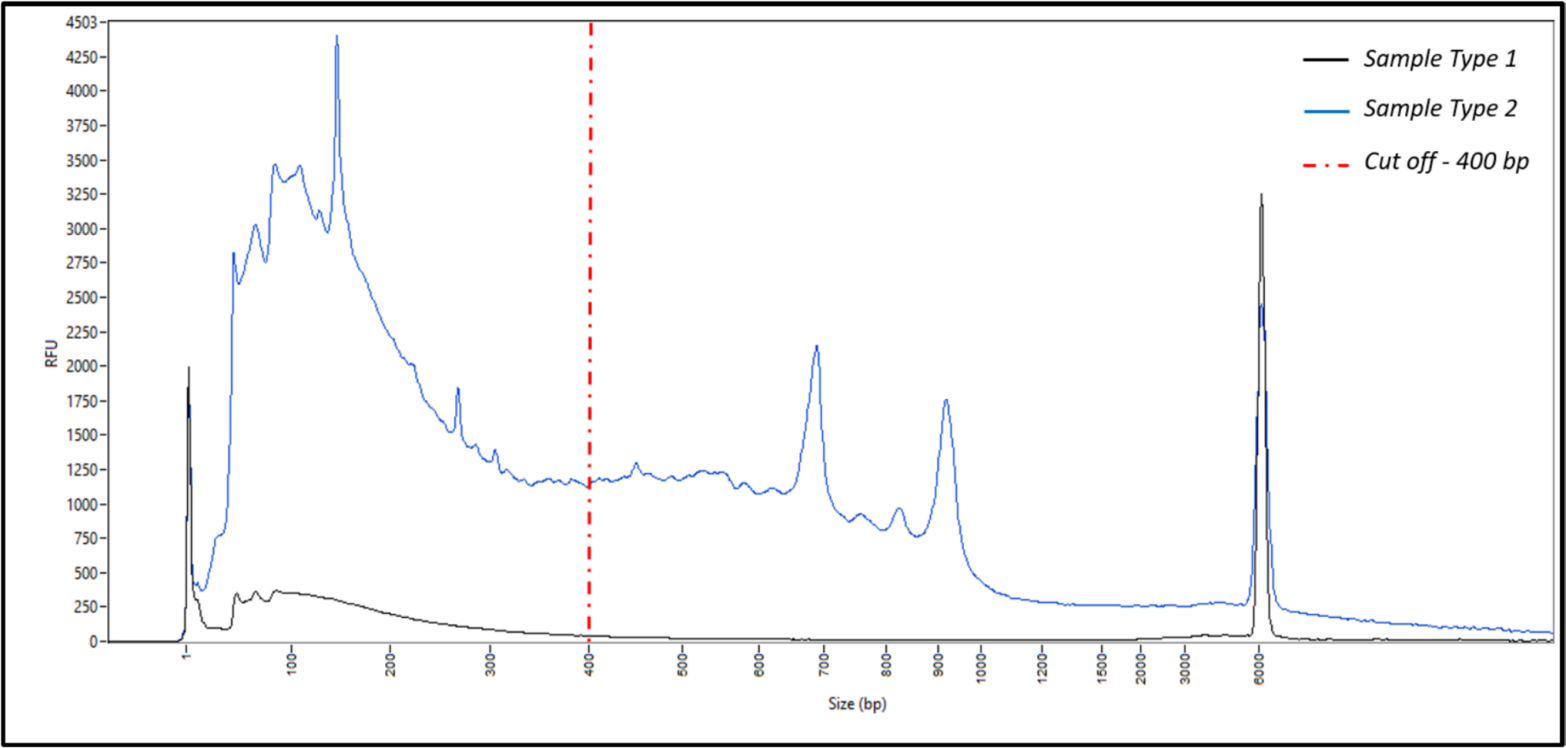
Representation of the femto pulse electrophoretic profiles from touch DNA casework samples divided into two groups: sample type 1 (black line), and sample type 2 (blue line).The red dashed line represents the cut-off at 400 bp, which could be used to distinguish between non-degraded samples (> 400 bp) and degraded samples (< 400 bp)

We then attempted to group the FPS profiles of these touch DNA samples according to specific criteria, including the overall appearance of their profile (e.g., peak balance, signal intensity), the presence of low molecular weight DNA fragments (visible below/above 400 bp in size), and the global DNA size range (distribution of fragments across the size spectrum). We tried to correlate this to the support used to collect the samples by visual examination by three experts, but without success. Yet, we could distinguish two main types of FPS profiles (Fig. 6): 75% of the traces showed a profile with a majority of DNA fragments of small size (< 400 bp) and low DNA amounts (sample type 1, a profile more similar to that of the model touch DNA), and 25% showed a profile with DNA fragments of larger size (> 400 bp) and/or significant amounts of DNA (sample type 2).

Although the FPS results suggested the presence of DNA at a concentration compatible with establishing a STR DNA profile, none of the samples provided a usable STR DNA profile (too partial to be included in the national database). Since the FPS detects all DNA (human and non-human), it was assumed that another source of DNA was present in the samples. While we can group similar femto pulse profiles, there is no correlation with the support of casework using only this technology. FPS is sensitive enough to detect DNA in every trace tested, but it doesn’t explain why these traces fail to provide a useful DNA profile. Further characterization is needed to understand the origin of these profiles and to identify those for which genetic profiling efforts could be made.

### Analysis of the presence of human DNA and fragmentation rate in DNA traces using *Alu* sequences

In order to determine why casework samples with apparently sufficient amounts of DNA did not result in usable DNA profiles, we evaluated the quantity and quality of human DNA present in the samples using primate-specific markers (*Alu* sequences, highly abundant in human DNA).

The calibration of the method used serial dilutions of NA12878 sample from 1 ng to 0.002 pg (1 ng, 100 pg, 10 pg, 1 pg, 0.1 pg, 0.01 pg, 0.002 pg), positive and negative controls (Supplementary material S4). As expected, no DNA amplification was observed in the negative controls or bacterial strains (Ct above 35 indicating non-specific amplification). Positive amplification was obtained with both *Alu115* and *Alu247* for human and macaque DNA. Ct values between macaque and human were similar at equivalent concentrations (100 pg/µl), indicating that the number of *Alu* sequences is similar between these two species with genomes of similar size.

#### Model samples

The five model tissues samples (blood, saliva, touch DNA sample, semen and vaginal swab) were tested with *Alu*-qPCR. The estimated amounts of DNA were equivalent to those previously obtained using a Qubit 3.0 fluorometer. The *Alu115*/*Alu247* ratio was equal to 1, suggesting that the DNA was not degraded as expected for these fresh model traces.

#### Casework samples

1 of the 100 DNA extracts was completely consumed by various tests to set up the different technologies used. We thereby applied this method to 99 casework samples. The results are shown in Table 1. 81 samples were amplified with the *Alu115* and *Alu247* primers, 2 samples were amplified only with the *Alu115* primers, and 16 samples were not amplified at all. Thus, the search for the presence of human DNA in the forensic traces was positive in 84% of the cases with an estimated mean concentration of DNA of 1.4 pg/µl with *Alu115* and 0.70 pg/µl with *Alu247*, indicating that in most samples the DNA was highly fragmented and therefore of very poor quality (Supplementary material S5). The ratio of non-human DNA to human DNA ranges from 1.3 to 23 000 with an average of 640 (non-human DNA was quantified by measuring the difference between total DNA obtained by FPS and by *Alu-*qPCR). Our results showed that non-human DNA was much more abundant in the samples analyzed, masking the human DNA concentration.

**Table 1:** Results of *Alu*-qPCR in the 100 casework samples

Alu qPCR results	Number of samples
Positive in *Alu115* and *Alu247*	81
Positive in *Alu115* but negative in *Alu247*	2
Negative in *Alu115* and *Alu247*	16
Insufficient material	1
Total of casework samples	100

The *Alu*-qPCR method used in this study was also compared to one of the commercial qPCR kits commonly used in forensic genetics: the Investigator® Quantiplex® Pro Kit (QPP, Qiagen), which is also routinely used at IRCGN. This kit not only measures total DNA but also assesses the state of degradation by targeting two fragments of different sizes, 91 bp and 353 bp, providing a degradation index based on the ratio between these fragments, which estimates the integrity of the DNA. The results of this comparison are shown in Supplementary material S5 and suggest that quantification with *Alu* sequences detects on average more DNA than the QPP Kit.

Morever, we applied the Index of Integrity (INTI) calculation to the 38/100 samples with STR profiles, albeit partial, and compared the results with those from *Alu*-qPCR and the QPP Kit. The analysis showed a correlation between low INTI values and higher DNA fragmentation, highlighting the convergence of INTI, qPCR methods, and FPS profile results in assessing DNA degradation in forensic samples. Supplementary material S5 presents the information on INTI and 1/INTI for the 38 samples.

In conclusion, human DNA was present in the majority (84%) of suboptimal casework samples, but might be in too small quantity to be analyzed by current technologies. Moreover, most visible DNA with the FPS might be non-human DNA.

### Analysis of the presence of microorganisms in DNA traces

To better characterize and understand the complexity of a suboptimal traces, the analysis of microbial DNA was conducted on six DNA touch casework samples representing different types of FPS profiles (Fig. 5). Human DNA was detected by *Alu*-qPCR in samples 2 and 3, whereas samples 1, 4, 5 and 6 were negative, indicating either the absence of human DNA or a level of degradation incompatible with PCR using these primers. These samples were amplified using two methods known as Nested and Fuhrman methods. Both methods were evaluated to determine the most appropriate for future forensic applications. Post-amplification DNA quantification results showed that DNA from microorganisms was present in all samples using the Nested method. On the other hand, only half of the samples were positive with the Fuhrman method (samples 1, 2, and 3).

**Fig. 7:**
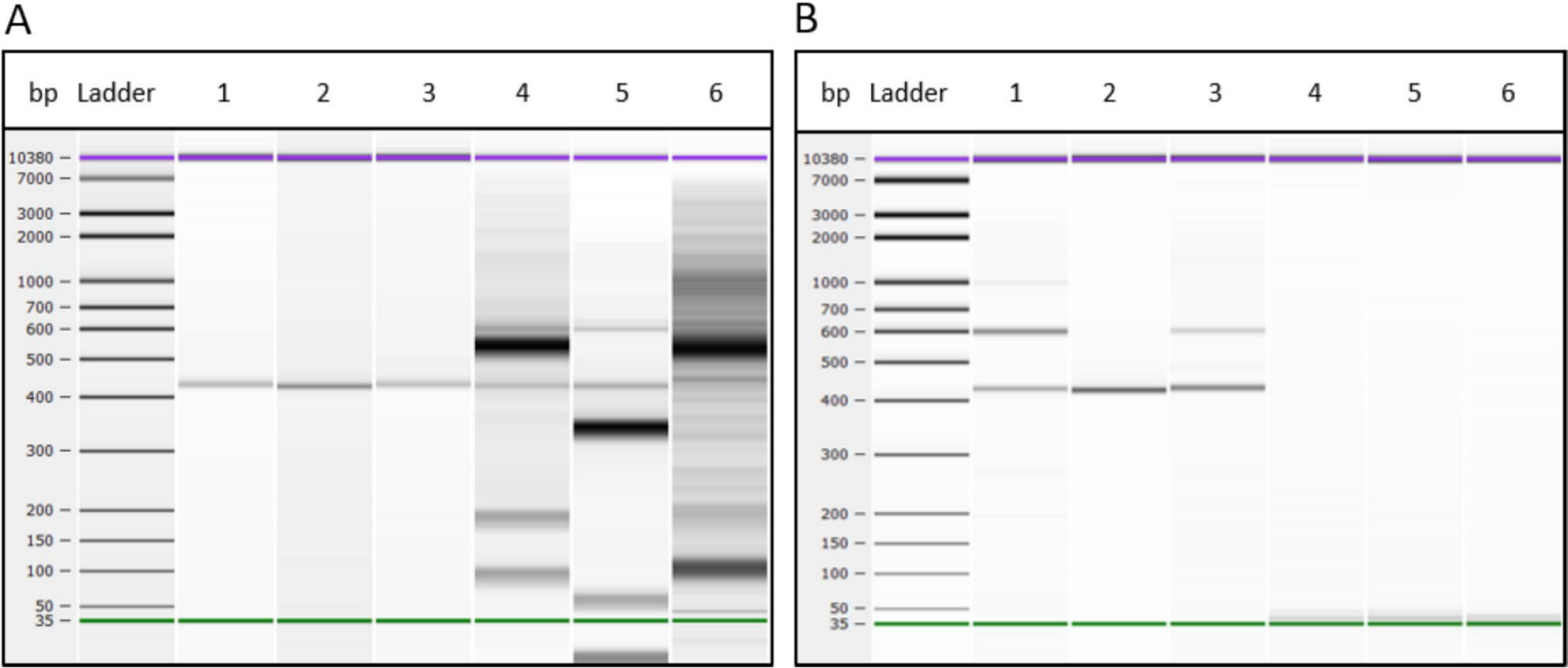
Results of the amplification of the V4 and V5 regions of 16S ribosomal RNA analyzed by electrophoresis using 2100 Bioanalyzer technology with (**a**) the Nested method and (**b**) the Fuhrman method. The band at 430 bp is specific to prokaryotes and the band at 600 bp is specific to eukaryotes. Non-specific amplification bands are present in samples 4, 5, and 6 with the nested method

The results obtained with the 2100 Bioanalyzer showed variations depending on the method used for amplification but were consistent (Fig. 7). Amplicons of 430 bp and 600 bp corresponded to prokaryotic (16S) and eukaryotic (18S) rRNA amplification, respectively. Samples 1, 2, and 3 were positive with both the Nested and Fuhrman methods (band at 430 bp). However, 2 out of 3 samples (samples 1 and 3) showed a band at 600 bp with the Fuhrman method. This sequence also cross-reacts with eukaryotes and was not visible with the Nested method since this method starts with a bacterial-specific amplification. Samples 4, 5, and 6 were negative with the Fuhrman method and gave multiple bands with the Nested method (specific for prokaryotes at 430 bp but with a lot of non-specific amplification).

**Fig. 8:**
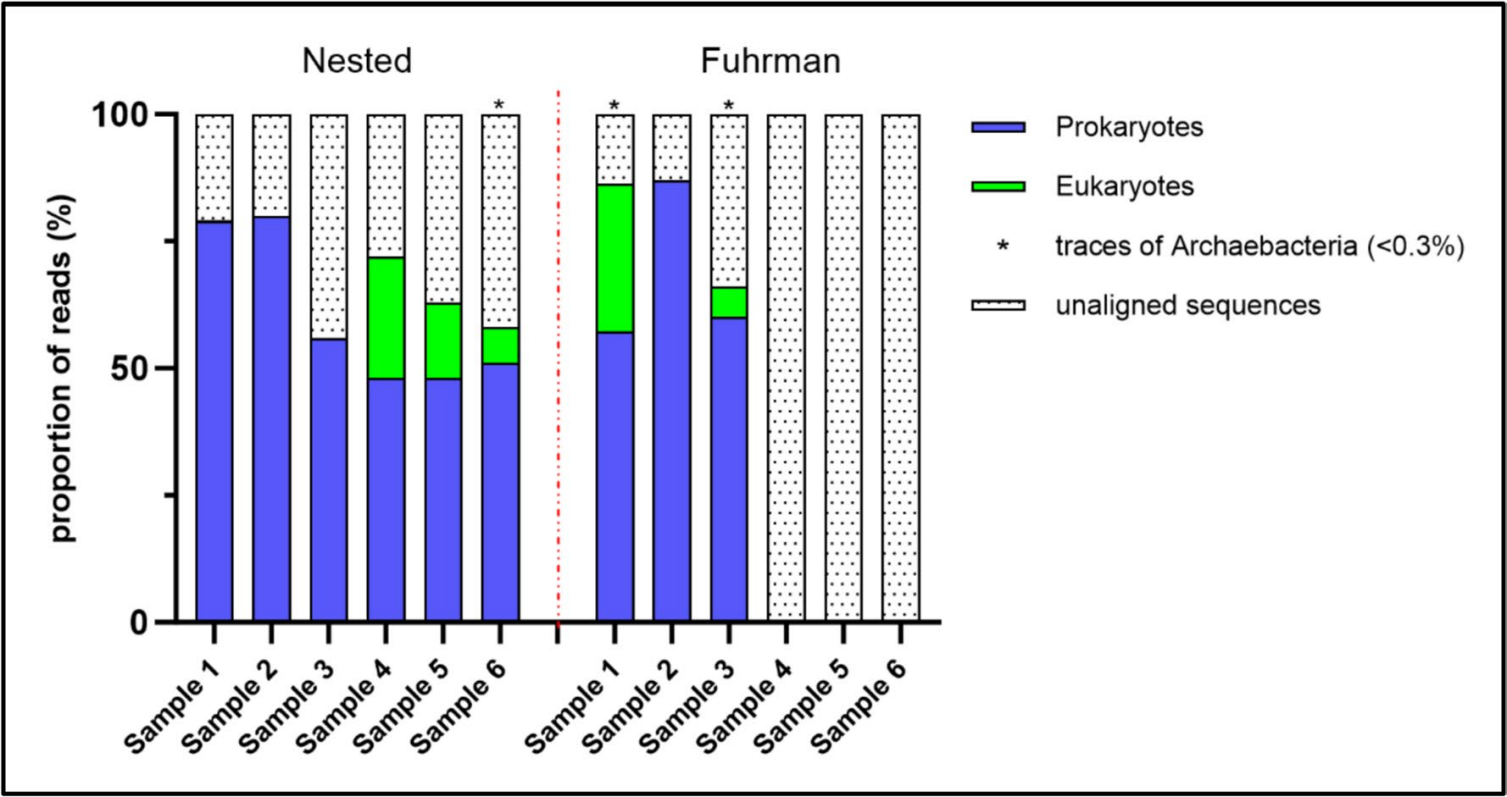
Results of the proportions of prokaryotes (blue), eukaryotes (green) and archaebacterial (*) with the Nested and Fuhrman method in six casework samples

The sequencing results are presented in Fig. 8. The proportion and contribution of prokaryotic and eukaryotic DNA in a sample were calculated from the number of reads obtained for each sample. Samples that were positive with both methods (samples 1, 2, and 3) contained a majority of prokaryotic DNA (bacteria), some eukaryotic DNA, and traces of Archaea in some samples. The sequenced eukaryotic DNA corresponded to two classes of fungi: *Ascomycota* and *Basidiomycota* [27]. Samples 4, 5, and 6, which were positive only with the Nested method, consisted of approximately 50% prokaryotic DNA (bacteria) and between 7 and 24% fungal DNA.

When we analyzed these data and their corresponding FPS profiles, we observed the following trends:

- the least degraded FPS profiles (with both small and large fragments) indicated the presence of prokaryotic DNA (samples 1 and 2);
- degraded FPS profiles (with only small fragments) did not yield any results with the Fuhrman method, but detected both prokaryotic and eukaryotic DNA with the Nested method (samples 5 and 6).
- moderately degraded FPS profiles (mostly small fragments but with some large fragments) showed that only one out of two samples yielded 16S rRNA results (samples 3 and 4). For this last category, predicting the presence of 16S rRNA from the FPS profile is challenging.

The sequencing data were further examined to identify the most abundant bacterial genus in these DNA traces. After classifying the sequence results by cluster, then by domain, microorganism species and finally by bacterial genus, 10 bacterial genera were identified as the most abundant in casework samples (Table 2). The results were equivalent for both the Nested and Fuhrman methods. The bacterial genera identified were present in different proportions depending on the sample. There were 5 bacterial genera that were present in all samples: *Sphingomonadales Sphingomonas*, *Cytophagales Hymenobacter*, *Rhizobiales Methylobacterium*, *Burkholderiales Massilia* and *Caulobacterales Brevundimonas*. Our results showed that each sample had a distinct microbial composition.

**Table 2:** Results of the 10 most abundant bacterial genera in suboptimal casework samples (in percentage)

Order	Genus	Sample 1		Sample 2		Sample 3		Sample 4	Sample 5	Sample 6
Method (N: Nested; F: Fuhrman)		*N*	F	*N*	F	*N*	F	*N*	*N*	*N*
*Sphingomonadales*	*Sphingomonas*	16.38	23.06	5.54	5.64	6.56	13.7	23.50	4.58	1.09
*Cytophagales*	*Hymenobacter*	12.07	10.22	0.01	0.07	8.11	5.01	2.64	0.61	0.44
*Micrococcales*	*Arthrobacter*	11.26	6.95	0	0	1.88	1.58	0.03	0.09	0.09
*Rhizobiales*	*Methylobacterium*	10.78	10.77	0.58	0.49	4.44	5.84	18.13	2.29	0.22
*Deinococcales*	*Deinococcus*	7.33	5.58	0	0	0	0	0.58	0	0.16
*Cytophagales*	*Spirosoma*	1.18	0.70	0	0	6.44	4.86	1.71	0.29	0.10
*Burkholderiales*	*Massilia*	0.88	0.10	6.31	6.94	0.21	0.32	5.70	3.51	1.70
*Rhodobacterales*	*Rubellimicrobium*	0.59	0.36	0	0	5.21	4.91	0.65	0	0.09
*Caulobacterales*	*Brevundimonas*	0.04	0.36	5.88	6.00	0.75	0.84	0.37	0.98	0.31
*Pseudomonadales*	*Acinetobacter*	0.01	0	15.95	18.44	1.34	1.28	0	0.16	8.90
Others		39,48	41.9	65.73	62.42	65.06	61.66	46.69	87.49	86.9
Total		100	100	100	100	100	100	100	100	100

This comparative analysis highlights the effectiveness of the Nested method in detecting microbial DNA in casework samples, making it a promising technique for future forensic investigations where human DNA may be absent or highly degraded.

## Discussion

This study focused on the characterization of forensic traces that do not provide interpretable genetic fingerprints using the gold-standard processes implemented in forensic laboratories. These traces represent about 66% of cases [28] and cannot be used today as evidence to confound criminals or help to identify victims. It is likely that the unusability of these traces is due to several limitations: the quantity and integrity of the DNA, the contamination by other non-human nucleic acids, and, in some cases, the presence of complex mixtures composed of one or more subjects. In optimal traces, mixtures can be analyzed today using dedicated tools (such as STRmix™), but this remains difficult when the DNA is present in low quantities and is degraded, and it was not the focus in this study. However, the increasing capability to analyze low-quantity DNA-containing traces will likely reveal that traces are more complex than initially thought. The aim of our study was to better characterize these suboptimal traces in order to 1) understand their composition and 2) define filtering criteria to determine which traces could potentially generate a usable DNA profile using improved technologies.

We first introduced the use of a highly sensitive electrophoretic detection method, the FPS, which detects DNA down to femtograms. After some improvements, FPS has successfully detected and quantified DNA in every sample tested, but it could not guarantee that the profile was of human origin. The visualization of DNA in forensic traces that have failed to generate a DNA profile, despite having an observable DNA quantity detected by the FPS method that should be sufficient for profiling, suggests the hypothesis of the presence of degraded DNA along with contaminating non-human DNA.

To objectify and quantify the presence and integrity of human DNA, we used the amplification of primate-specific *Alu* sequences. Human DNA was identified in 84% of the traces, with degradation observed in about half of the samples. These data suggest that the failure to obtain a profile in about 66% of the collected traces is not due to the total absence of human DNA, but rather a limited amount and/or a degradation state incompatible with STR amplification. *Alu*-qPCR is therefore able to detect human DNA in cases that have failed to generate a usable DNA profile using current technologies. It is useful for identifying human DNA-containing traces that could benefit from future improved technologies managing traces with low and degraded DNA, such as sequencing [29].

To further understand the differences between the *Alu* and the QPP methods used, we conducted a more in-depth investigation into the QPP kit. This kit uses a target called 4NS1C, the use of which for quantification and degradation analysis is protected by a Qiagen patent (US20140234834A1). Mapping of 4NS1C on the CHM13v2.0 version of the human genome identifies 21 copies spread across 8 chromosomes, including 10 copies on chromosome 7. In comparison, the human genome contains over one million copies of Alu sequences spread across all chromosomes, making Alu sequences a potentially more appropriate element for analyzing degradation throughout the entire human genome.

Last, the INTI method can be applied but it requires genotyping data, whereas qPCR and FPS do not. This could help avoid unnecessary tests on samples that are not worth the trouble and economically more interesting.

From the FPS and *Alu*-qPCR experiments, the respective contributions of human and non-human DNA could be deduced. In 99% of traces, the amount of human DNA is much less abundant in percentage than non-human DNA. These results indicate that in order to valorize these traces, it is necessary to develop other technologies that can 1) miniaturize the process to cope with small amounts of DNA, and 2) take advantage of degraded DNA, characterized by short sequences, for example by using polymorphic markers detectable in short DNA sequences, such as SNPs [29].

Finally, we analyzed the contamination of the traces with microorganisms by sequencing the bacterial metagenomes of some samples, whose FPS profiles were representative of the entire set of 100 casework samples available for analysis. Microorganisms were identified in all these traces. The 10 bacterial genera identified in the majority of the traces varied in proportion. In particular, each trace had a different composition, which could be used as an additional signature. These microorganisms can originate from several sources, such as the microbiota of the individual who left the trace or from bacteria present in the environment.

To further interpret these findings, it is essential to evaluate whether the microorganisms identified in our samples are typical of the tissue or environment associated with the traces. In our study, the 10 bacterial genera isolated from samples 1 to 6 are mainly environmental bacteria (soil, water, plants). Nevertheless, since some genera have specific characteristics in relation to their habitat this information can be valuable. For example, Arthrobacter is usually associated with soil, particularly hydrocarbon-contaminated soils, but has also been isolated from wastewater and animals, while Hymenobacter and Deinococcus is renowned for its extreme resistance to radiation and is typically found in radioactive or desert-like environments [30]. These characteristics suggest that many of the microorganisms in our study likely originated from the surrounding environment rather than from the human body. However, certain species like Sphingomonas paucimobilis and Massilia varians have been found in humans, often in the context of infections [31, 32] and as such could help further identify a suspect since some of these infections are easily recognizable. For the genus Massilia, up to 5 species have been recovered from clinical specimens [32]. However, the ribosomal RNA analysis we have carried out generally identifies only the genus (in our case, Sphingomonas or Massilia for example) but not the species (paucimobilis or varians for example.) Identification of the species will therefore require additional sequencing which could be reserved for the most serious cases.

This duality in microbial origin underscores a common forensic challenge: distinguishing between environmental and human microbiomes, particularly in samples exposed to a variety of external factors. As Satari et al. (2020) [33] highlighted, bacterial communities can evolve from a human-associated microbiota (e.g., oral bacteria) into an environmental bacteriome over time, which could further explain the predominance of environmental bacteria in our samples (e.g., Sphingomonas, Deinococcus, Hemenobacter). Their study on chewed gum has showed how human-associated microbiota rapidly transition into environmental bacteriomes after environmental exposure, which can further complicate the identification of the bacterial source in crime scene samples. Indeed, this study reveals that the oral microbiota present in gums after being chewed, characterized by the presence of Streptococcus spp. or Corynebacterium spp., evolves in a few weeks to an environmental bacteriome represented by Acinetobacter spp, Sphingomonas spp. and Pseudomonas spp. These findings suggest that while most bacteria identified in our samples are mostly environmental in origin, the contribution of human microbiota cannot be ruled out.

Ultimately, a specific microbiota could contribute to criminal identification by revealing bacteria unique to an individual or linked to a particular environment. Pathogenic or non-pathogenic bacteria specific to a suspect might reveal information about the individual’s health status [34] thus complementing traditional forensic data derived from DNA traces. The unique microbial signatures found in these samples could also serve as geographic markers, connecting a suspect or object to a particular location, as evidenced by previous forensic soil microbiome studies [35].

Although larger studies are needed to further investigate the data from this work, the analysis of microorganisms in these samples highlights the potential for microbiota analysis in forensic science [36, 37], beyond its more classical use to determine time of death [38]. This analysis underscores the potential of integrating microbiome data with existing forensic methods. By combining bacterial metagenomic data with traditional human DNA profiling, forensic scientists could gain a more holistic understanding of both the biological and environmental origins of traces found at crime scenes. This “multidimensional” approach could improve the accuracy of investigations and strengthen the evidence, particularly in cases where DNA traces alone are insufficient to establish a clear link between an individual and a crime scene.

In our study, it was observed that traces collected quickly after deposition (model samples) exhibited relatively low microbial presence based on the FPS profiles obtained. This is likely due to minimal exposure to environmental factors, which limits bacterial colonization. On the other hand, casework samples, collected after a longer period, showed a significant increase in microbial presence as suggested by the FPS profile and the results obtained with 16S rRNA. This aligns with findings that environmental exposure promotes the accumulation of bacteria, both from human interaction and environmental contamination. As discussed previously, over time, microbial communities diversify, and environmental bacteria begin to dominate the microbial profile of a trace.

By combining these observations, the possible increase in bacterial DNA in older traces, the potentially higher total DNA content in the femto pulse profiles, and the progressive degradation of DNA, we can hypothesize that it may be feasible to estimate the time elapsed since deposition in complement to earlier study [39]. This estimation could be based on 1) the amount of bacterial DNA in the trace, 2) its femto pulse profile (highly degraded or poorly degraded), and 3) the bacterial identification in the trace. Further research would be essential to validate this approach, particularly to account for the numerous environmental factors that could influence DNA degradation and microbial population dynamics.

The use of advanced technologies in forensic settings raises economic considerations, particularly in terms of long-term sustainability. The cost of the methods used in this study must be compared to traditional tools to assess their viability. The cost of Femto Pulse technology combined with *Alu*-qPCR is similar to that of a standard qPCR analysis using commercial kits (around $5 per sample), while offering better resolution for suboptimal traces, making it a viable alternative. For 16S rRNA sequencing, costs are around $30 per sample, depending on the provider.

## Conclusion

In this work, we have shown that the use of three technologies (FPS-based resolutive electrophoresis, *Alu*-qPCR, and bacterial sequencing) allowed to characterize the DNA content in forensic casework samples that have failed genetic profiling. In our experience in France, about 66% of the traces collected in forensic practice do not produce a DNA profile [29] that can be transferred to the national database. Our work indicates that human DNA is present in at least 84% of these samples. Small amounts of DNA, combined with a level of degradation that makes it impossible to use standard technologies, are the main reasons for these failures. Bacterial DNA predominates in the majority of these traces, which complicates the sorting of the traces according to their total DNA content and may represent an additional opportunistic signature that could be exploited independently. Despite these challenges, the improvement of existing technologies [40], or the introduction of new ones, offers the prospect of exploiting these traces, particularly those associated with the most serious crimes. Several promising directions emerge 1) the miniaturization of processes to concentrate DNA in smaller volumes, 2) the adaptation of PCR protocols to cope with limited amounts of DNA, and 3) the introduction of SNP markers detectable by sequencing in degraded DNA where STR genotyping is impossible. Developing these directions will maximize the potential of even suboptimal traces, thereby rethinking our approach to DNA profiling in forensic science.

## Supplementary Information

Below is the link to the electronic supplementary material.

ESM 1 Dotplot of DNA concentrations from all model samples across different tissues and supports: (a) blood, saliva, and touch DNA; (b) semen samples and vaginal swabs. (PNG 50.5 KB)

High Resolution Image (TIF 217 KB)

ESM 2 (PNG 18.9 KB)

High Resolution Image (TIF 89.0 KB)

ESM 3 Electrophoretic profiles of DNA from blood and saliva. Results from different supports are shown for two donors using the ultra sensitivity NGS Kit. The results between the different donors are similar. The observed differences are due to the quantity of DNA. (PNG 323 KB)

High Resolution Image (TIF 223 KB)

ESM 4 Femto pulse electrophoretic profiles of saliva DNA at different reaction volumes. Final reaction volumes were tested: 12 µl (blue line), 10 µl (red line), 8 µl (black line), and 6 µl (black line). The DNA of interest is not visible when the final volume is less than 10 µl. The results obtained with a volume of 10 µl allow better visualization of the sample compared to 12 µl. (PNG 103 KB)

High Resolution Image (TIF 30.3 KB)

ESM 5 Results of amplification curves for Alu DNA markers using (a) Alu115 and (b) Alu247 primers. Serial human DNA dilutions (inputs ranging from 1 ng, 100 pg, 10 pg, 1 pg, 0.1 pg, 0.01 pg, and 0.002 pg) are shown in black. The amplification of macaque DNA (0.1 ng/µl) is shown in green, the 3 bacterial samples are shown in blue, and the amplification of H2O and Tris–EDTA (negative controls) are shown in red. The results show that the macaque DNA is positive for Alu and the bacterial samples are negative. (PNG 951 KB)

High Resolution Image (TIF 155 KB)

ESM 6 DNA concentration results from 100 suboptimal casework samples. The table shows, for each sample, the concentration obtained by qPCR with the primers Alu115 and Alu247, the fragmentation rate (the ratio between Alu115 /Alu247), the average concentration obtained from the Alu115 and Alu247 primers, the concentration obtained using the Investigator® Quantiplex® Pro Kit (QPP) with 91 bp and 353 bp fragments, the Qiagen fragmentation indicator, the fragmentation rate QPP (the ratio between QPP 91 bp / QPP 353 bp), the average concentration obtained from both QPP frangments, index of integrity (INTI), 1/INTI, the total DNA concentration obtained by the FPS, the non-human DNA concentration (the difference between the total DNA and the human DNA), and finally the ratio between the non-human DNA and the human DNA. Sample 69 has no results because there was not enough material to perform the analyses. (XLSX 23.1 KB)

## Data Availability

All data generated or analyzed during this study are included in this published article and its supplementary materials.
