# Supplementary figures and images for: Characterization of challenging forensic DNA traces using advanced molecular technologies
Source: Int J Legal Med. 2025 Mar 1;139(4):1511–27. doi: 10.1007/s00414-025-03448-8 (PMC12170681; doi:10.1007/s00414-025-03448-8)

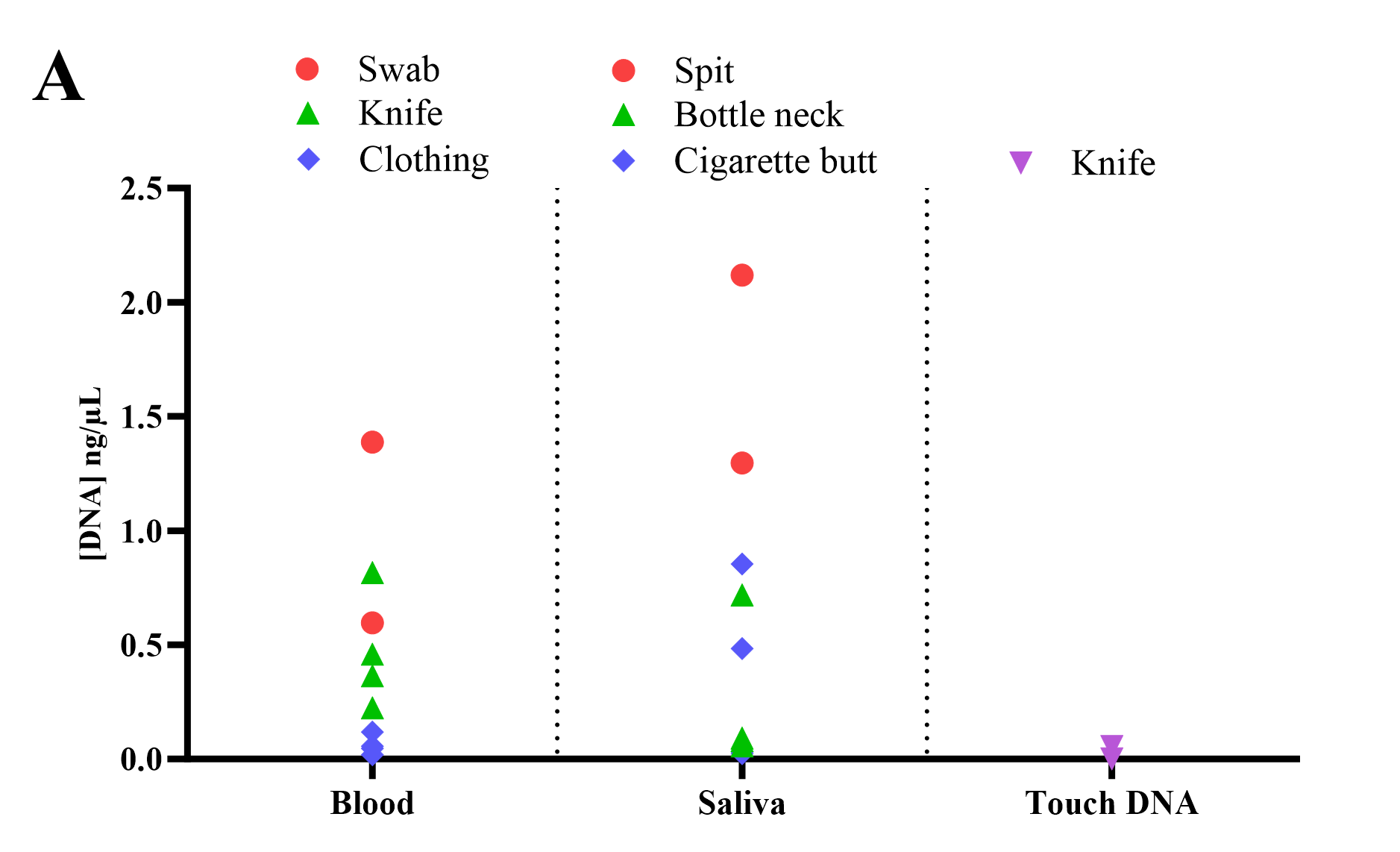

Supplement: Supplementary file 1 — Dotplot of DNA concentrations from all model samples across different tissues and supports: (a) blood, saliva, and touch DNA; (b) semen samples and vaginal swabs. (PNG 50.5 KB) [file 414_2025_3448_Fig9_ESM.png]

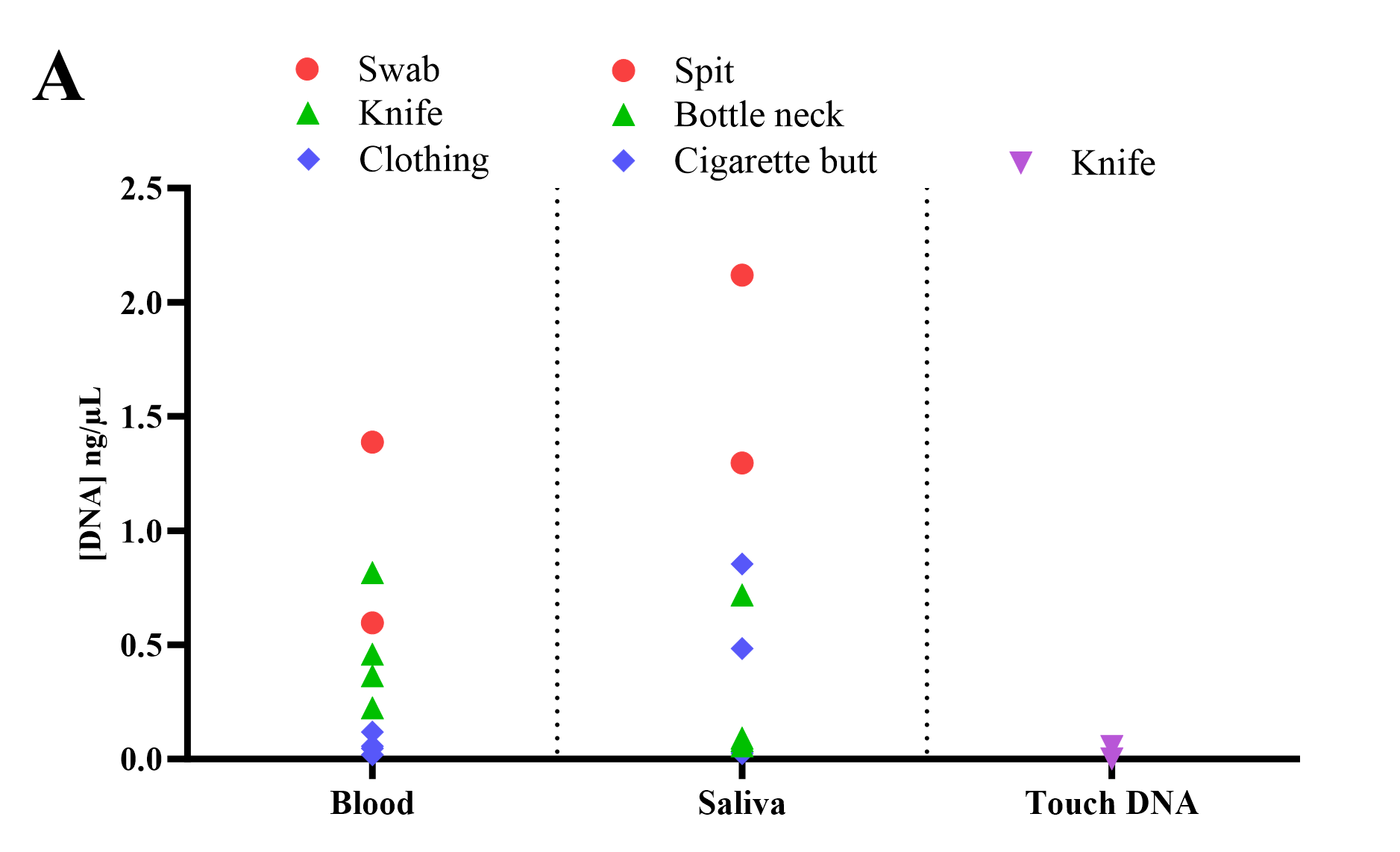

Supplement: Supplementary file 2 — High Resolution Image (TIF 217 KB) [file 414_2025_3448_MOESM1_ESM.tif]

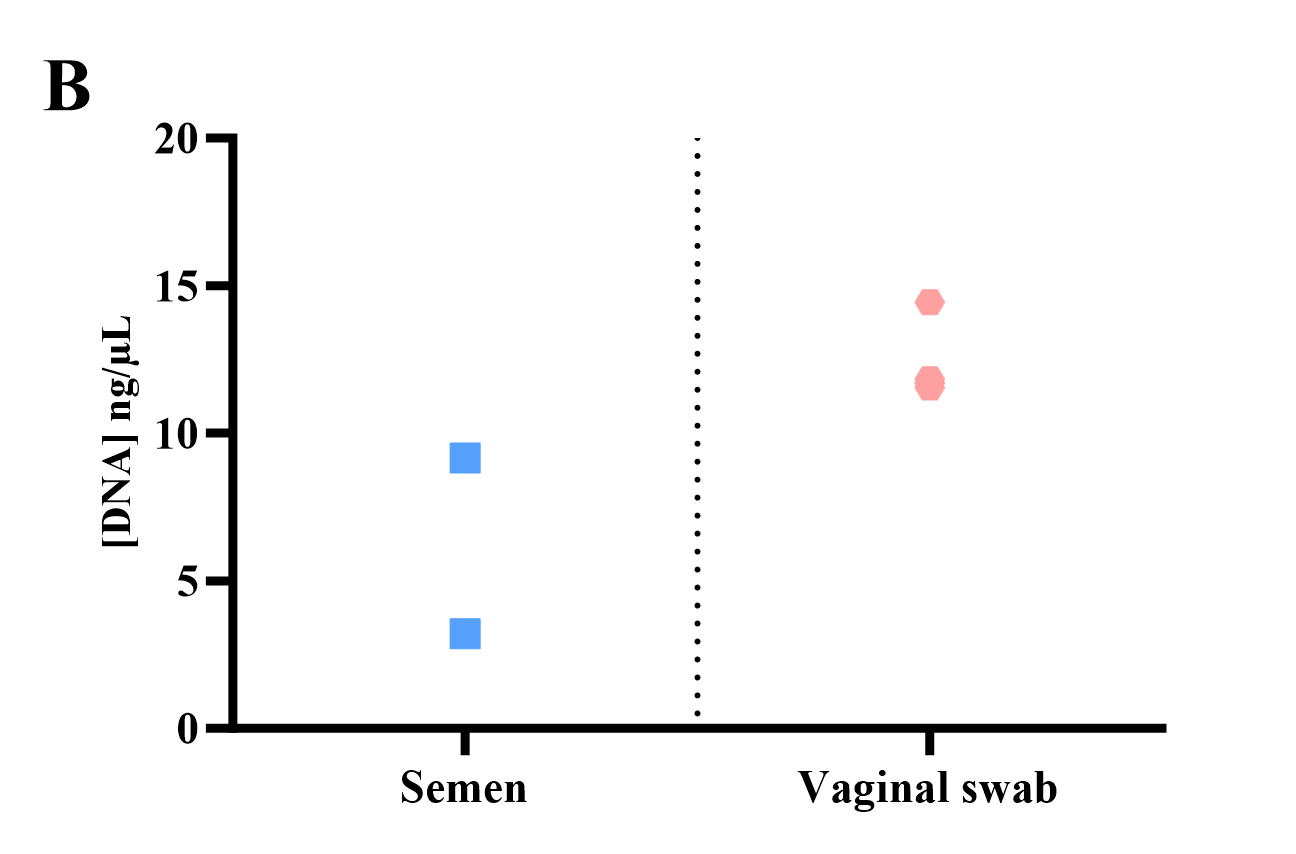

Supplement: Supplementary file 3 — (PNG 18.9 KB) [file 414_2025_3448_Fig10_ESM.png]

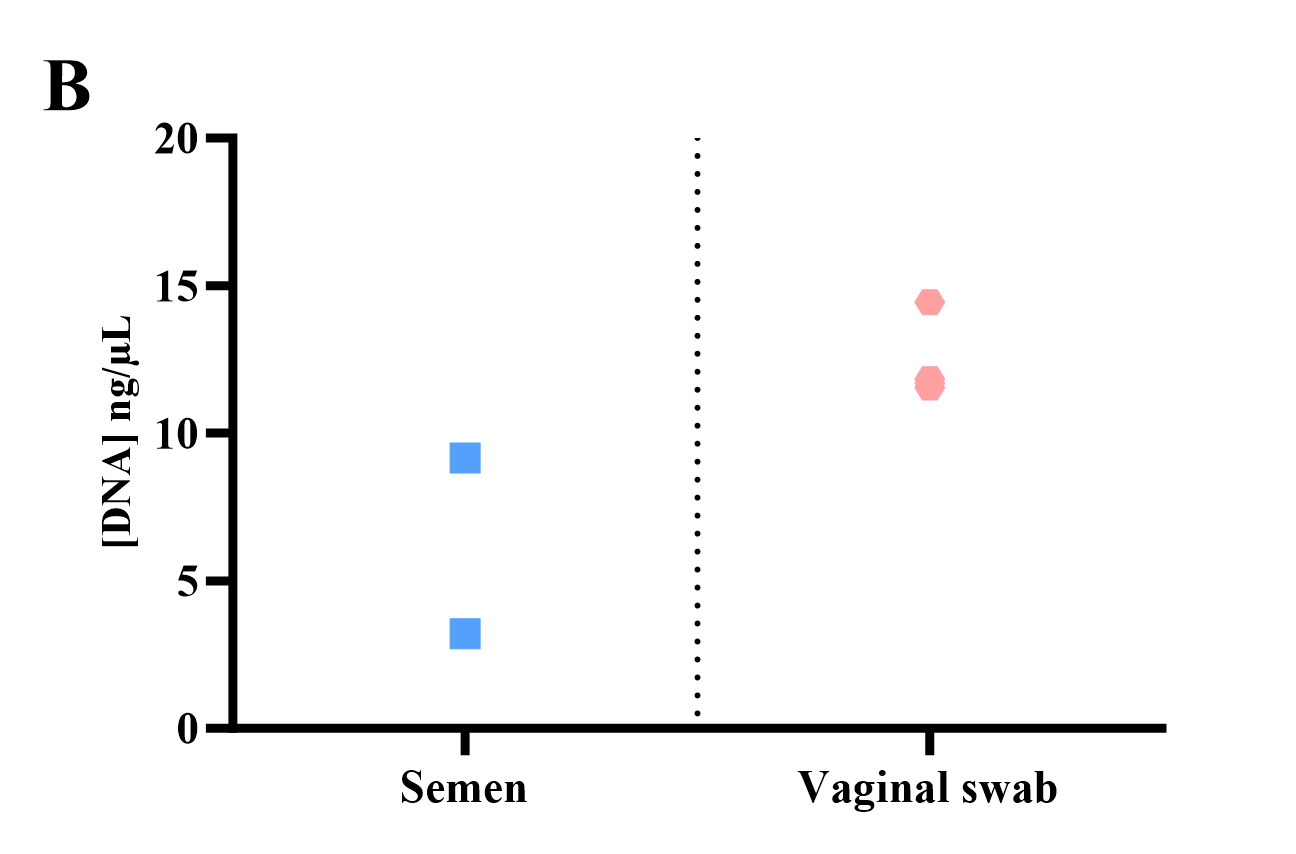

Supplement: Supplementary file 4 — High Resolution Image (TIF 89.0 KB) [file 414_2025_3448_MOESM2_ESM.tif]

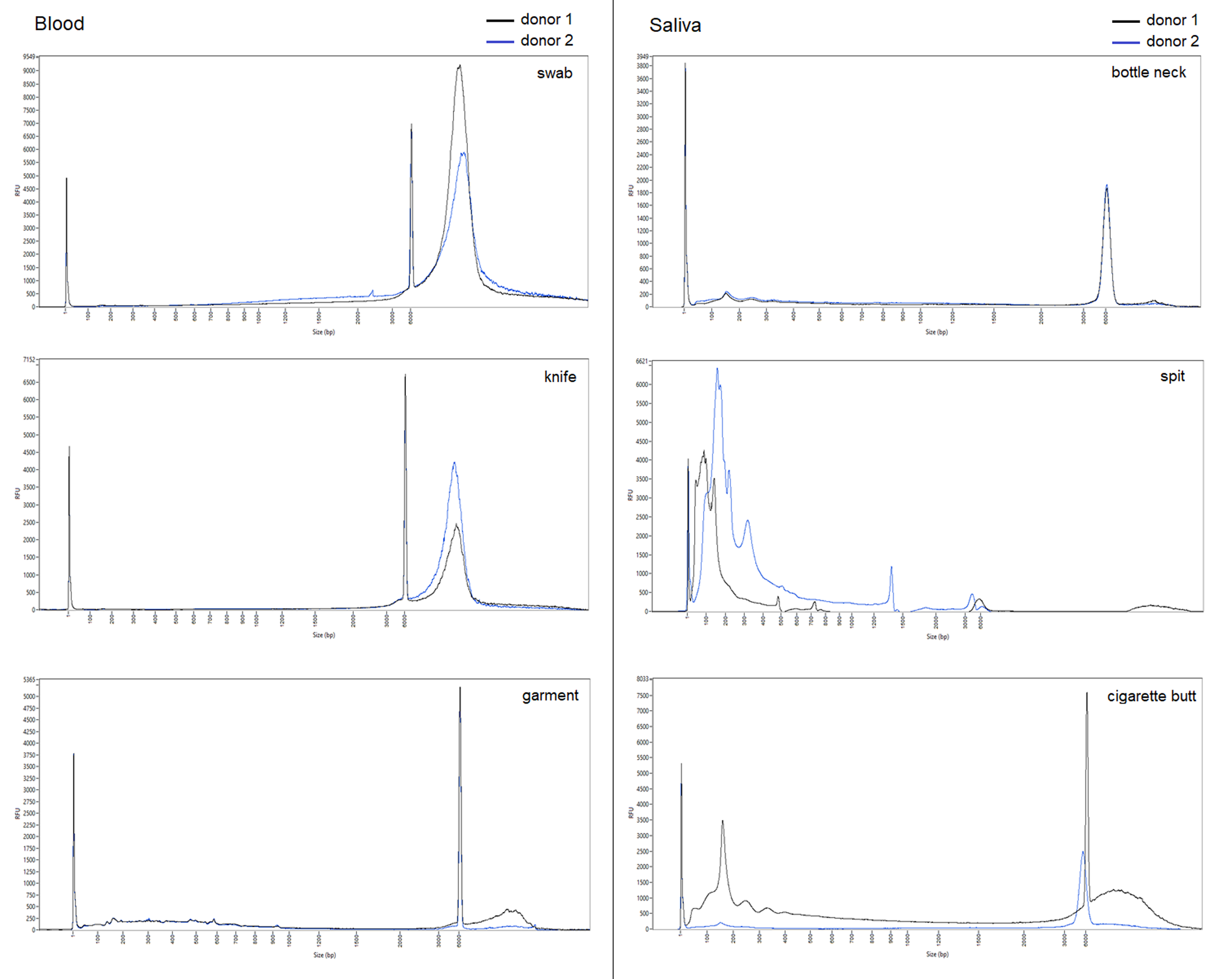

Supplement: Supplementary file 5 — Electrophoretic profiles of DNA from blood and saliva. Results from different supports are shown for two donors using the ultra sensitivity NGS Kit. The results between the different donors are similar. The observed differences are due to the quantity of DNA. (PNG 323 KB) [file 414_2025_3448_Fig11_ESM.png]

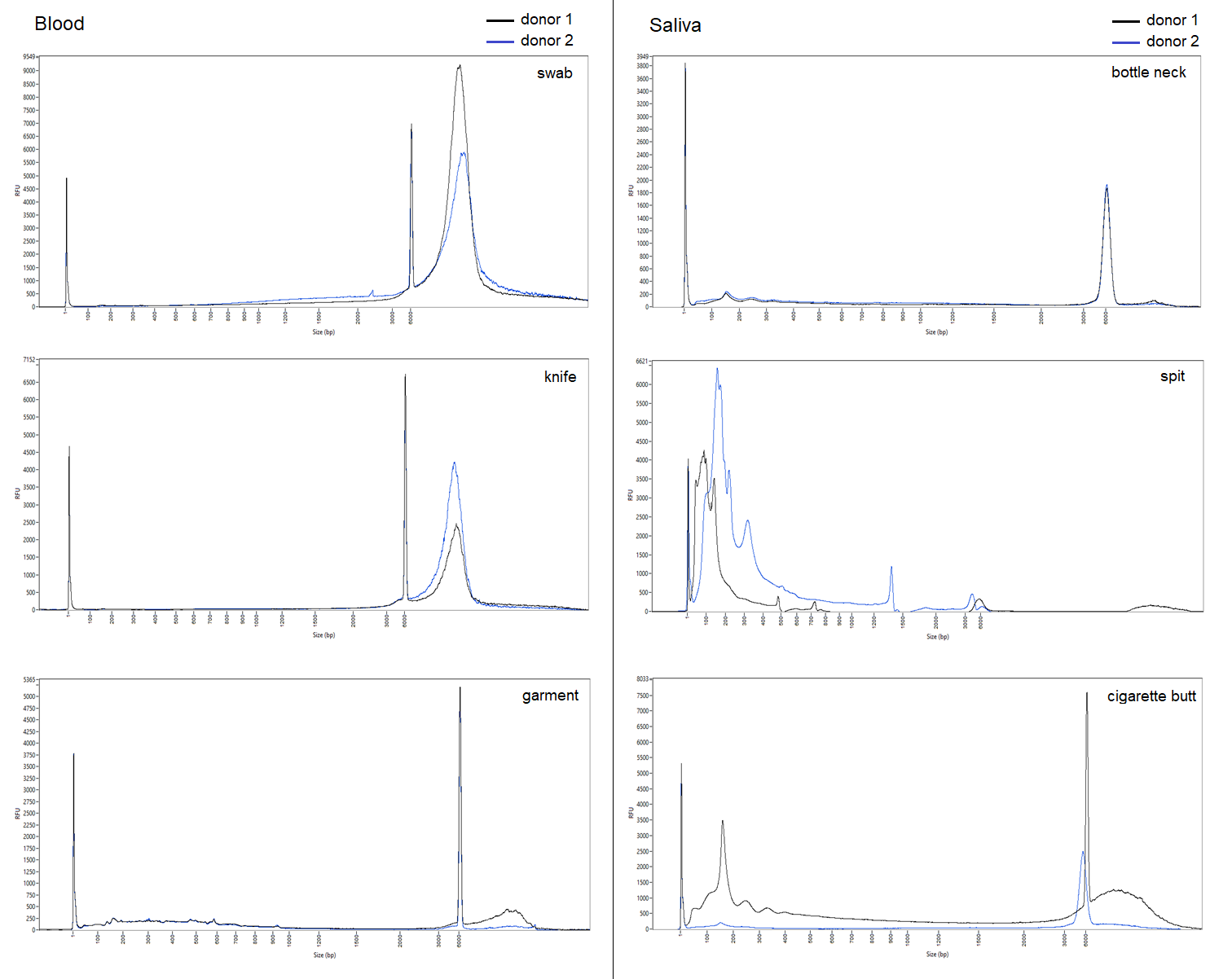

Supplement: Supplementary file 6 — High Resolution Image (TIF 223 KB) [file 414_2025_3448_MOESM3_ESM.tif]

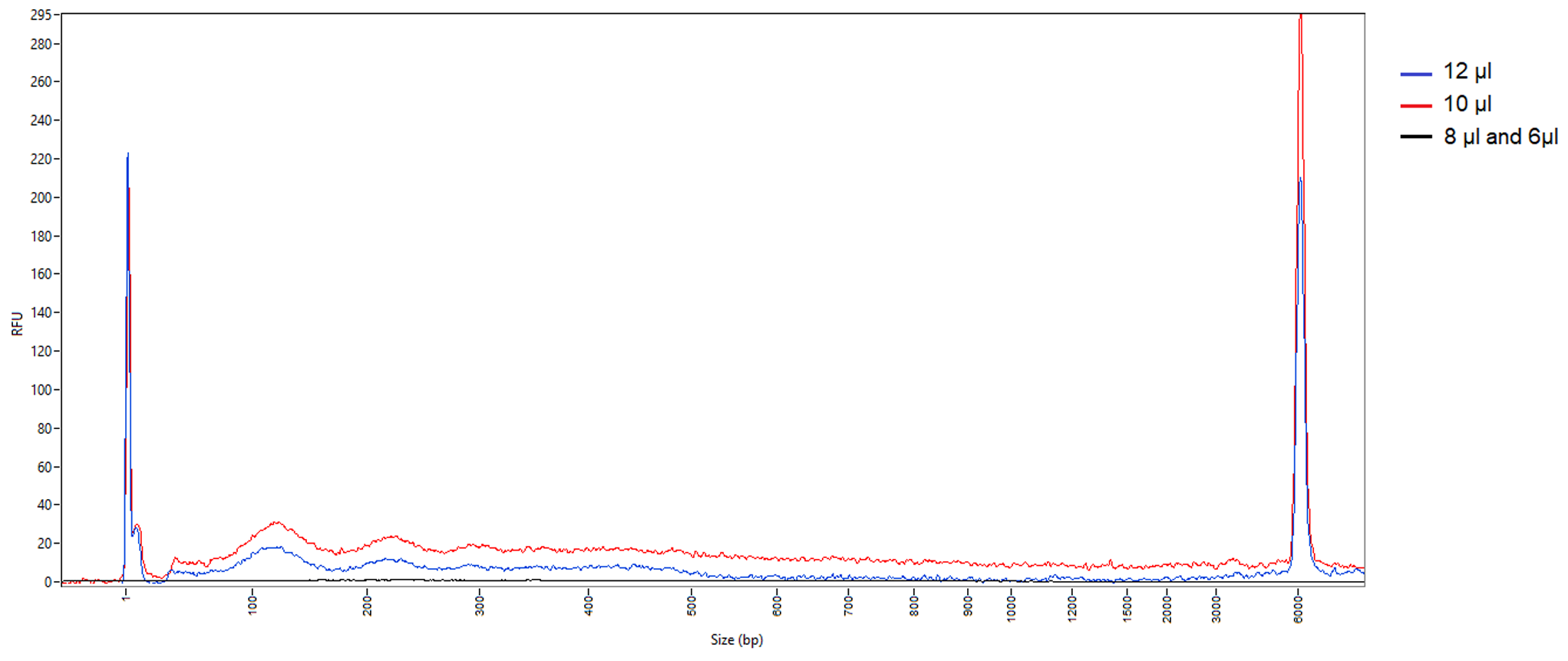

Supplement: Supplementary file 7 — Femto pulse electrophoretic profiles of saliva DNA at different reaction volumes. Final reaction volumes were tested: 12 µl (blue line), 10 µl (red line), 8 µl (black line), and 6 µl (black line). The DNA of interest is not visible when the final volume is less than 10 µl. The results obtained with a volume of 10 µl allow better visualization of the sample compared to 12 µl. (PNG 103 KB) [file 414_2025_3448_Fig12_ESM.png]

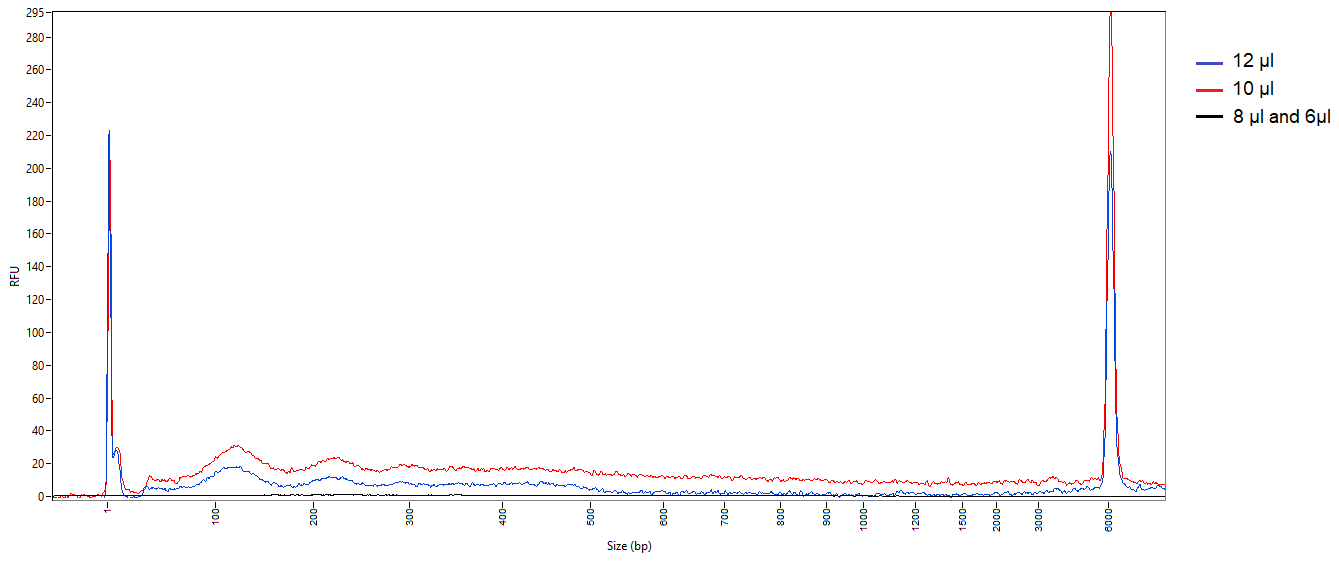

Supplement: Supplementary file 8 — High Resolution Image (TIF 30.3 KB) [file 414_2025_3448_MOESM4_ESM.tif]

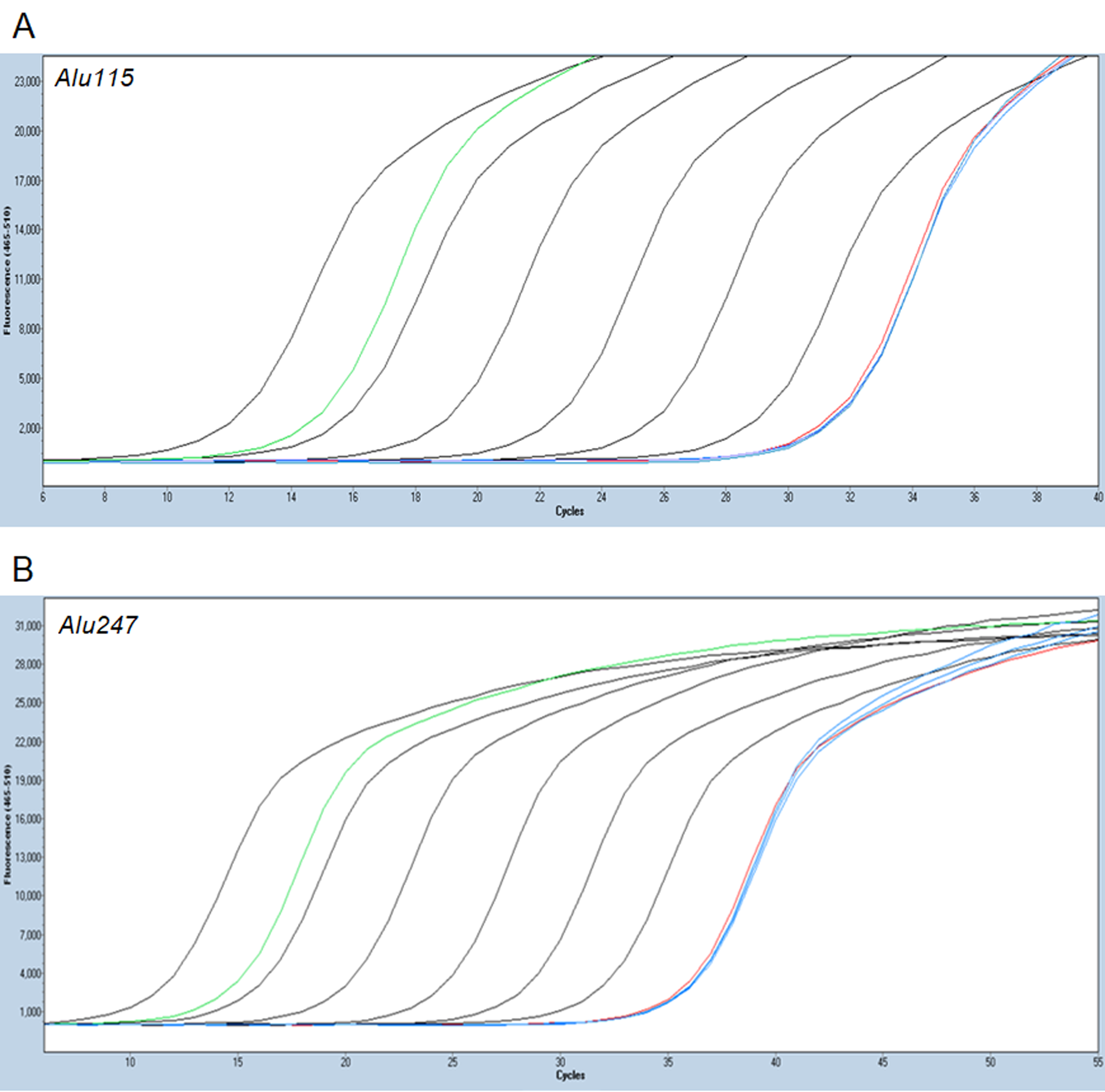

Supplement: Supplementary file 9 — Results of amplification curves for Alu DNA markers using (a) Alu115 and (b) Alu247 primers. Serial human DNA dilutions (inputs ranging from 1 ng, 100 pg, 10 pg, 1 pg, 0.1 pg, 0.01 pg, and 0.002 pg) are shown in black. The amplification of macaque DNA (0.1 ng/µl) is shown in green, the 3 bacterial samples are shown in blue, and the amplification of H2O and Tris–EDTA (negative controls) are shown in red. The results show that the macaque DNA is positive for Alu and the bacterial samples are negative. (PNG 951 KB) [file 414_2025_3448_Fig13_ESM.png]

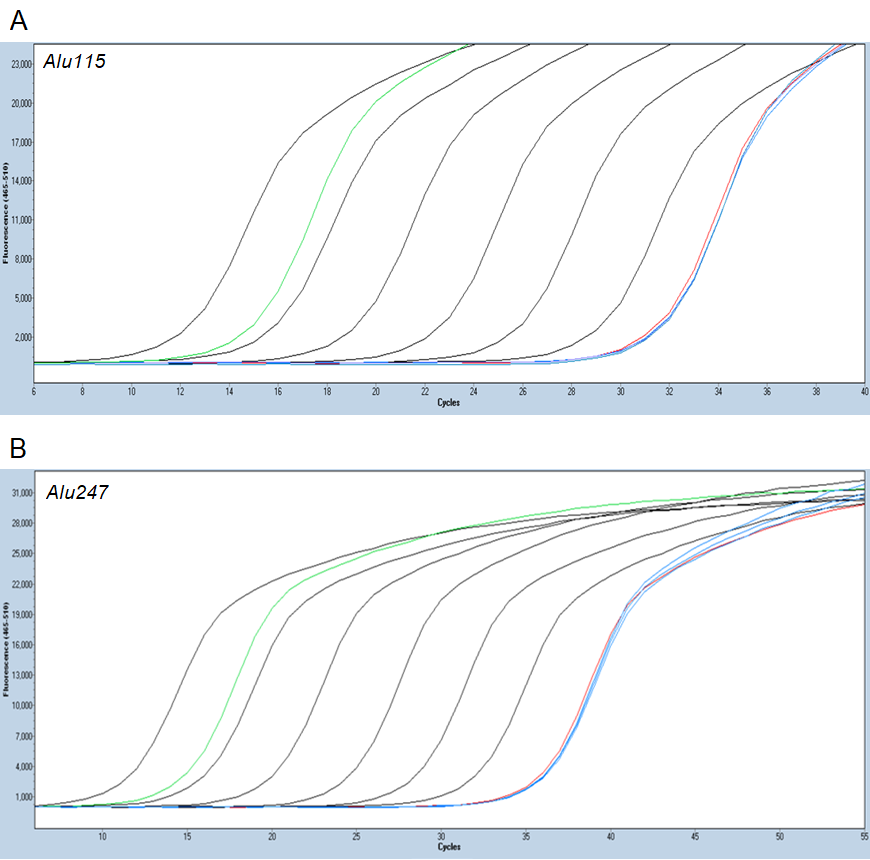

Supplement: Supplementary file 10 — High Resolution Image (TIF 155 KB) [file 414_2025_3448_MOESM5_ESM.tif]
